# Optimal Approach for Signal Detection in Steady-State Visual Evoked Potentials in Humans Using Single-Channel EEG and Stereoscopic Stimuli

**DOI:** 10.3389/fnins.2021.600543

**Published:** 2021-02-18

**Authors:** Zoltan Derzsi

**Affiliations:** ^1^Department of Psychology, New York University Abu Dhabi, Abu Dhabi, United Arab Emirates; ^2^Institute of Neuroscience, Newcastle University, Newcastle upon Tyne, United Kingdom

**Keywords:** EEG, frequency tagging, SSVEP, phase coherency, stereograms, disparity

## Abstract

In EEG studies, one of the most common ways to detect a weak periodic signal in the steady-state visual evoked potential (SSVEP) is spectral evaluation, a process that detects peaks of power present at notable temporal frequencies. However, the presence of noise decreases the signal-to-noise ratio (SNR), which in turn lowers the probability of successful detection of these spectral peaks. In this paper, using a single EEG channel, we compare the detection performance of four different metrics to analyse the SSVEP: two metrics that use spectral power density, and two other metrics that use phase coherency. We employ these metrics find weak signals with a known temporal frequency hidden in the SSVEP, using both simulation and real data from a stereoscopic apparent depth movement perception task. We demonstrate that out of these metrics, the phase coherency analysis is the most sensitive way to find weak signals in the SSVEP, provided that the phase information of the stimulus eliciting the SSVEP is preserved.

## 1. Introduction

While acquiring an EEG signal is easy, the difficulty of recording decent quality signal cannot be overstated: since the data acquisition equipment is working with very small voltages, it is very susceptible to various external (electrical grid, smartphones, etc.) and internal (eye blinks, vascular pulse, etc.) noise sources, which are often several times more powerful than the signal intended to be measured. Unfortunately, various signal processing techniques can clean up an existing recording only to a certain degree, which may not be sufficient in applications where the signals are exceptionally weak. Despite these problems, EEG remains popular since it has excellent temporal resolution and it is non-invasive: in the clinic it may be used to detect anomalous oscillations in patients with epilepsy or migraine (Camfield et al., 1978; Adeli et al., 2003), it may be used for characterizing a transient or steady-state response in the brain (Ciganek, 1961; Norcia et al., 2015), or may be used in Brain-Computer Interface (BCI) applications (Bayliss and Ballard, 2000; Nakanishi et al., 2018).

One of the oldest applications for EEG is to investigate the response of a brain when exposed to a transient stimulus. In the 1960s, this was done with presenting flashes of light, and the EEG signal was recorded by taking a photograph of the EEG trace displayed on an oscilloscope (Ciganek, 1961). Since the recorded signals were very noisy, the experiment was repeated several dozen times, and the corresponding films were overlaid on each other. After this operation, the magnitude of the noise is reduced, and a clearer transient response, what we now call a flash-evoked potential or Event-Related Potential (ERP) is revealed. The modern-day equivalent of overlaying exposed and developed films on top of each other is averaging the EEG signal in the time domain: after capturing the response for a flash is presented in each trial, it is possible align each trial's recording to the time of the flash, and averaging the signals to reveal the ERP. This time-averaged signal then can be evaluated against a comparison standard (signal absent, healthy controls, etc.) later-on in the analysis.

Based on the same principle, it is possible to not only investigate the transient response, but the steady-state response of the brain as well: a more sophisticated example is the “frequency tagging” technique, where instead of a single transient stimulus, a continuous periodic stimulus is used. Using the flash example above mentioned earlier, this would mean using flickering light instead of presenting a single flash. Provided that the light conditions are carefully chosen (Herrmann, 2001; Norcia et al., 2015), and are kept constant throughout the trials, the stimulus elicits a detectable neural response, and if there are enough trials recorded, the amplitude of the frequency or frequencies corresponding to the stimulus will elevated in the spectrum of the SSVEP. The elevated frequencies may not always be the same as the temporal frequency of the stimulus (Hébert-Lalonde et al., 2014): it may be a harmonic (Norcia and Tyler, 1984) or if several temporal frequencies are used at the same time, they may produce intermodulational products (Baitch and Levi, 1988): the sum and difference of these frequencies, or an arbitrary combination of these. SSVEPs may be studied with techniques other than EEG as well, such as Magnetoencephalography (MEG) (Srinivasan et al., 1999) or functional Magnetic Resonance Imaging (fMRI) (Boremanse et al., 2013) as well.

To analyse SSVEPs, the signal is converted between the time and frequency domains with a time-frequency transform, which is typically the Fourier transform (Norcia et al., 2015), or alternatively, the continuous wavelet transform (Daubechies, 1990; Adeli et al., 2003; Wu and Yao, 2007) may be used. There is a free and open-source software implementation of both of these, and they are part of a larger package called EEGLAB (Delorme and Makeig, 2004).

With the use of the Fourier transform, if *S*_*k*_(*t*) is the EEG trace recorded at a particular channel on the *k*th trial where the stimulus was presented, we can define the Fourier component *V*_*k*_(*f*) at the frequency *f* as:

(1) $$V_{k}(f)=\frac{1}{T_{w}}\int_{0}^{T_{w}}e^{2\pi ift}S_{k}(t)dt$$

where *T*_*w*_ is the temporal window which contains the SSVEP and is being used for the Fourier-transform. It is worth noting that Equation (1) is for analogue signals that are continuous in time. In modern computer systems, the signals are sampled at a rate that is at least twice more than the maximum intended temporal frequency to be recorded. In EEG, this sampling frequency ranges between 250 Hz and 1 kHz. On the sampled signal, which is now discrete in time, it is still possible to execute the Fourier transform, which is typically done by the Fast Fourier Transform (FFT) algorithm (Cooley and Turkey, 1965). The FFT produces a number of components (or “bins”), that are corresponding to discrete temporal frequencies. They contain the spectral power and phase of a small band of these frequencies determined by the ratio of the sampling frequency and the number of samples in the FFT window. To find which component corresponds to a particular temporal frequency in the analogue signal, the following equation may be used:

(2) $$n_{c}=\frac{f_{x}}{\left[\left(f_{s}/2)/(n_{w}/2\right)\right]}+1;\;where\;n_{C}\in N$$

*f*_*x*_ is the temporal frequency in question, *f*_*s*_ is the frequency the EEG signal was sampled at, *n*_*w*_ is the FFT window size, which is the number of samples the FFT algorithm worked with. The +1 term is there to add the offset for the component corresponding to the temporal frequency of 0 Hz, which is also called the “Direct Current” (DC) component. As the result of the FFT is discrete in frequency, *n*_*c*_ can only be a natural number and the equation's result should be rounded to the nearest integer. Once the correct Fourier component is identified using this equation, we can use the same signal processing steps as we would for an analogue signal. The Fourier-component is a complex number, and has an amplitude and phase component.

**Figure 1:**
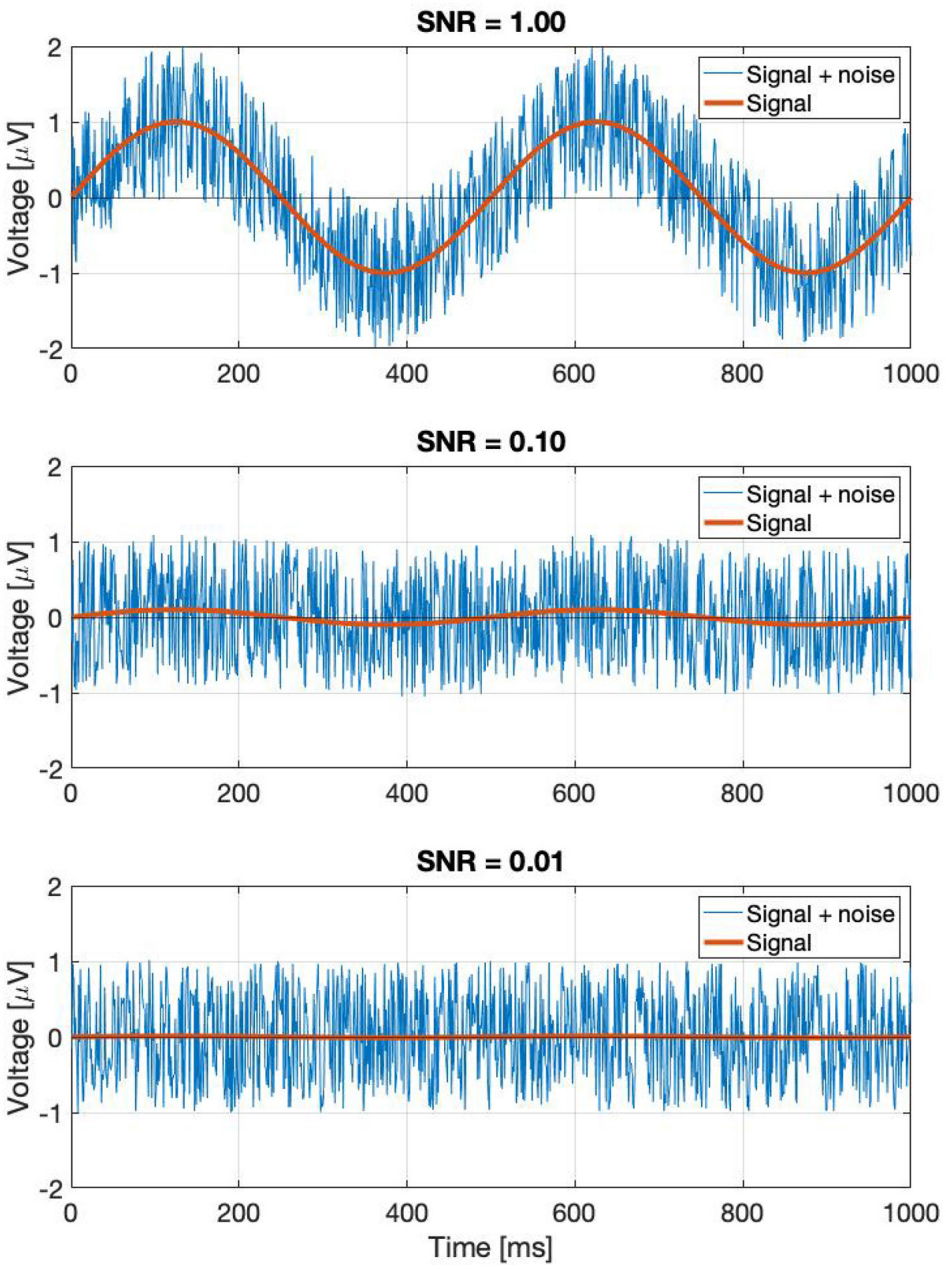
If we take a complex Fourier component *V*_*k*_(*f*) from a trial, it can be represented as a vector. As this is an EEG signal, it will be naturally noisy. The noise is a vectorial sum of two orthogonal components: the amplitude noise which only affects the length of the vector, and phase noise, which changes the phase angle of the vector, without affecting its amplitude.

We assume that the SSVEP in the EEG recording in the presence of a periodic stimulus is a linear combination of the “signal” that we want to measure (the neural response to the periodic stimulus), and the “noise” which represents the measurement of every other source contained in the EEG trace. If both the signal and the noise are small enough to not cause non-linear distortion, we can express them as:

(3) $$V_{k}=\left[\left(A_{0}+\alpha_{k})exp(i\phi_{0}+i\zeta_{k}\right)\right]+\left[\left(N_{0}+\beta_{k})exp(i\xi_{k}\right)\right]$$

where the first term represents the Fourier component of the signal which is the response to the periodic stimulus; and the second term is the response due to the noise in the EEG trace. *A*_0_ and *N*_0_ are the mean values of the signal and noise amplitudes, respectively across *k* trials. α_*k*_ and β_*k*_ are trial-dependent. ϕ_0_ is the phase of the signal that is elicited by the periodic stimulus at the frequency *f*. Without loss of generality, we have defined the mean phase in the noise as being zero: < ζ_*k*_ >=< ξ_*k*_ >= 0, where <> denotes the average over the *k* trials. For clarity we have dropped the (*f*) throughout, but both terms in this equation depend on the frequency *f*. It is applicable for both analog signals by using *f* directly, and for digital signals by finding the corresponding Fourier component using Equation (2). Note that the amplitude noise components (α_*k*_; β_*k*_) and the phase noise components (ζ_*k*_; ξ_*k*_) are orthogonal. Their effect on the signal *V*_*k*_ is illustrated in Figure 1.

### 1.1. Different Methods for Estimating the SNR

From Equation (3), we have seen that even a single Fourier-component is a consist of several noise terms, and unfortunately we do not have access to the particular values of these noise terms following a time-frequency transform. Since the definition of noise is very broad and effectively covers any signal or phenomena that is unwanted, different estimation methods exist in the literature. Provided that we have an understanding of the neural mechanism we are investigating, we can select a smaller band in the spectrum. For example, in a study where we have a good understanding of the neural mechanism tested, it is possible to anticipate which temporal frequencies will be present in the SSVEP. In studies where FFT is used to analyse the spectrum of the SSVEP, an acceptable approach is to take the neighboring 10–20 Fourier components surrounding the temporal frequency of interest, and calculate its average value. Then, the SNR may be estimated by calculating the ratio of the values of the of the Fourier component of interest, and the noise level. This was done in several studies (Srinivasan et al., 1999; Cottereau et al., 2011; Alonso-Prieto et al., 2013; Boremanse et al., 2013), and with this method, it is impossible to detect signals that are below the noise.

This method works best if the spectrum used for calculating the noise levels are clear enough, and we know precisely which temporal frequency we are expecting the signal to be present in the SSVEP. Unfortunately, in some cases we may not have such luxury: the noise may not be pure white or pink noise, or perhaps other oscillations may be present in the SSVEP that may not be related to the stimulus at all. In these cases, this SNR estimation method may not be reliable.

**Figure 2:**
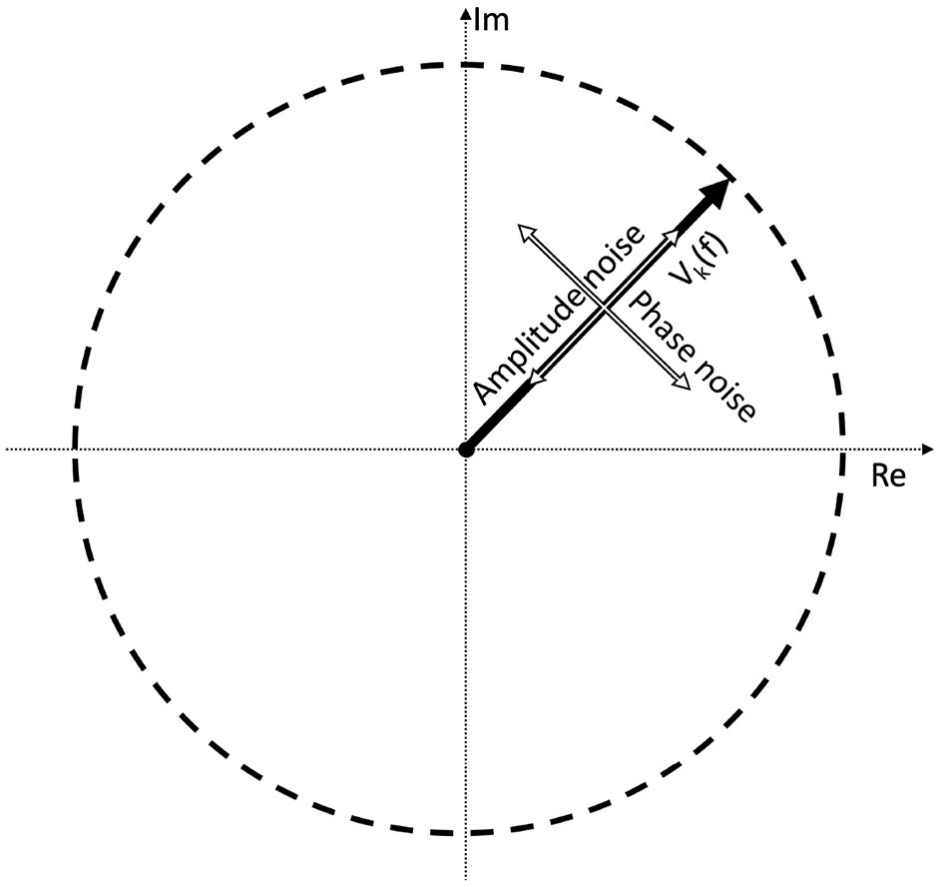
A demonstration of noisy signals in the time domain. From the top to the bottom, the signal is faded a hundred times.

Perhaps a better approach is to go back to the original definition: noise is every signal we don't want in our recording. The signal we are investigating is weak, and is buried in the EEG trace in the time domain. If the signal was strong, we would be able to find it just by looking at the EEG signal itself in the time domain. In the time domain, the SNR can simply be calculated by the taking the ratio of the peak levels, in a similar way how the *A*_0_ and *N*_0_ terms play a role in Equation (3). Since the SSVEPs are usually invisible in a single trial, we can assume that they are several times below the noise level. A few example representations of SNRs in the time domain are shown in Figure 2. Every subsequent signal processing step, such as filtering or averaging across trials is considered to be part of the detection process. Estimating in the SNR in the frequency domain is not as straightforward as in the time domain, since the noise power depends on the spectral bandwidth, and if discrete time signals are used, the values of the Fourier components will additionally depend on the ratio of the FFT window length and sampling frequency as well.

### 1.2. Detecting Weak Signals in the SSVEP

It is possible to express various metrics that can be used to evaluate the SSVEP and detect the signal.

**Figure 3:**
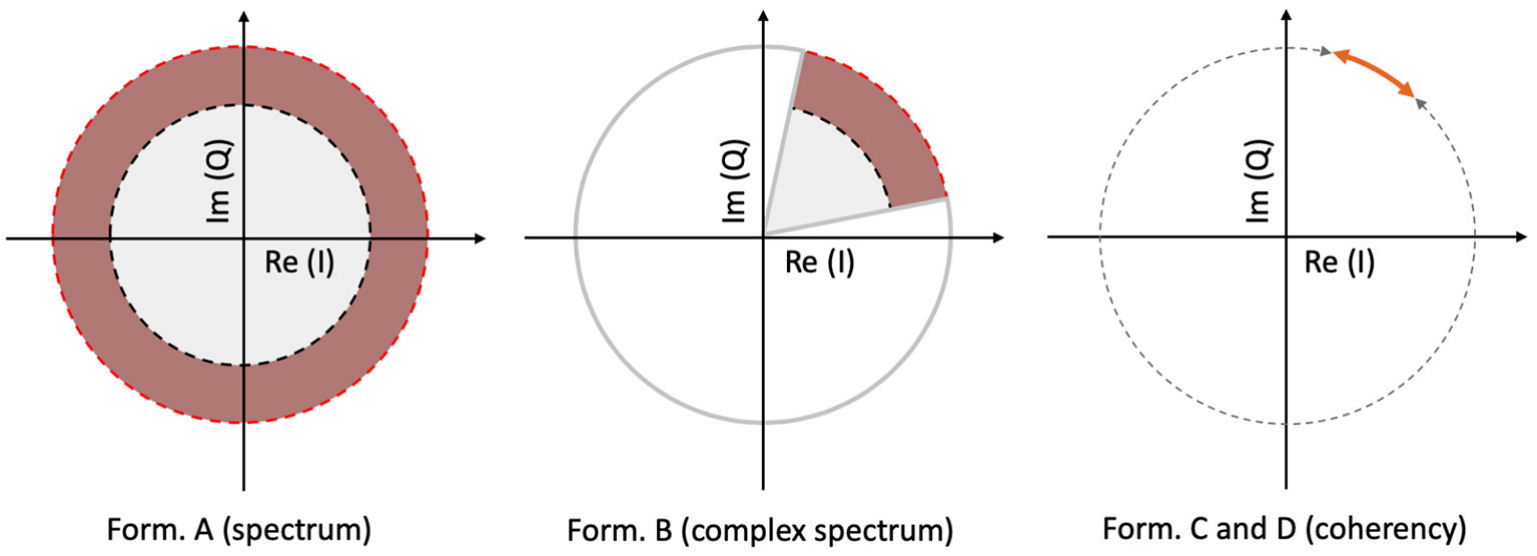
A visual illustration of how each metric operates, for a single frequency, in a vectorial form. In the left plot, the spectrum (formula A in Table 1) only detects the signal, when more than 95% of the samples are inside the shaded annulus, implying that this Fourier component's spectral power is significantly elevated. The complex spectrum (formula B in Table 1, and in the middle plot) is similar to formula A, but with an added phase criterion: the signal is only detected if 95% of the samples are inside the partial annulus; the Fourier component not only has to be significantly elevated, but also needs to be in a particular phase angle range as well. With coherency (right plot, formula C and D in Table 1), the signal only gets detected when 95% of the normalized vector samples are forming a small enough distribution with respect to the entire circle (orange line with respect to the gray dashed line). It doesn't matter where they actually are on the circle, as long as their spread is sufficiently low.

The spectrum is calculated by taking the scalar means of the amplitudes of the Fourier components for each temporal frequency, across all presentations of the stimulus. Formula A is used in Table 1 to calculate this metric. Since calculating the spectrum ignores the phase angles altogether, only the amplitude terms *A*_0_ and α_*k*_, *N*_0_ and β_*k*_ play a role in Equation (3). This is visually represented in the left plot of Figure 3: our signal is successfully found, when the 95% of k vectors are within the shaded annulus. The width of the annulus is proportional to the trial-dependent terms α_*k*_ and β_*k*_ in Equation (3). This formula represents the oldest and most straightforward approach. It is used in many studies, such as Norcia et al. (2015), Hébert-Lalonde et al. (2014), Scherbaum et al. (2011), Gruss et al. (2012), Kamphuisen et al. (2008), Kamphuisen et al. (2008), Skrandies and Jedynak (1999), Baitch and Levi (1988), Alonso-Prieto et al. (2013), Panicker et al. (2011), Rossion et al. (2012), Wu and Yao (2007), Mun et al. (2012), Gruss et al. (2012), and Rideaux et al. (2020). The spectrum can also be used as a control measure or comparison standard, to demonstrate some other technique. A few examples are: Hakvoort (2001) and Lin et al. (2006), where they used spectrum to demonstrate the superiority of multi-channel correlation analysis; and Nakanishi et al. (2018), where they used spectrum to demonstrate the effectiveness of extracting the task-related independent components of the EEG signal.

**Table 1:** Some metrics that can be extracted from an SSVEP.

**Name**	**Formula**	**Used in studies**
Spectrum	$A\left(f\right)=\frac{1}{n}\overset{n}{\underset{k=1}{\sum }}|V_{k}\left(f\right)|$	Norcia et al. (2015), Hébert-Lalonde et al. (2014), Scherbaum et al. (2011), Gruss et al. (2012), Kamphuisen et al. (2008), Skrandies and Jedynak (1999), Baitch and Levi (1988), Hakvoort (2001) as a control; Lin et al. (2006) as control; Alonso-Prieto et al. (2013), Panicker et al. (2011), Rossion et al. (2012), Wu and Yao (2007), Nakanishi et al. (2018) as control; Mun et al. (2012), Gruss et al. (2012), Rideaux et al. (2020), and many many more.
Complex spectrum	$B\left(f\right)=\frac{1}{n}|\overset{n}{\underset{k=1}{\sum }}V_{k}\left(f\right)|$	Norcia and Tyler (1984) with additional filtering in the time-domain; Cottereau et al. (2011) and they kept the values in a vectorial form; Boremanse et al. (2013) used formula A, but they averaged in the time domain first; Johansson and Jakobsson (2000)
Coherency 1	$C\left(f\right)=|\frac{\overset{n}{\underset{k=1}{\sum }}V_{k}\left(f\right)}{\overset{n}{\underset{k=1}{\sum }}|V_{k}\left(f\right)|}|$	Norcia and Tyler (1984)
Coherency 2	$D\left(f\right)=\frac{1}{n}|\overset{n}{\underset{k=1}{\sum }}\frac{V_{k}\left(f\right)}{\left|V_{k}\left(f\right)\right|}|$	Mitra and Pesaran (1999), Kamphuisen et al. (2008), Derzsi (2017), and the experiment in this paper

*V_k_(f) is the input Fourier component recorded by a particular channel at frequency f, in the kth trial across n trials*.

An other possibility is to take the vector average to calculate the complex spectrum, i.e., to average the complex Fourier components and then calculate the magnitude at the last step to get the result. This is done with formula B in Table 1. Assuming each trial consists of an integer number of stimulus cycles, this is equivalent to joining all the trials together in the time domain and computing the Fourier amplitude of the result. This metric takes both the amplitude and the phase (*A*_0_ and *N*_0_; ϕ_0_ in Equation 3, respectively) into account. In the middle plot of Figure 3, the complex spectrum is represented as a partial annulus: not only the 95% of the k vectors have to have a certain length to detect our signal, but they have to be grouped around a certain phase angle ϕ_0_. Similarly to the spectrum, the width of the partial annulus is dependent on the amplitude components, but additionally the angle of the partial annulus is proportional to the trial-dependent variations (ζ_*k*_ and ξ_*k*_ terms in Equation 3) of the phase components. With additional filtering in the time domain, formula B was used in the spectral analysis part of Norcia and Tyler (1984). While, Boremanse et al. (2013) used formula A in their study, they averaged the signal in the time domain first, thus effectively used formula B. Except for the missing final step, Cottereau et al. (2011) used formula B, but they kept the final result in vectorial form. Johansson and Jakobsson (2000) purely used formula B.

The third and fourth metrics are the two interpretations of the inter-trial phase coherency (ITC, or simply “Coherency”), and both formula C and D in Table 1 can be used to calculate it. The coherency metric gets rid of the amplitude component along with its per-trial variations by normalizing the length of the *V*_*k*_ vectors in Equation (3) to unity across trials, and only the phase information is preserved. From Equation (3), only the trial-dependent phase angle terms ζ_*k*_ and ξ_*k*_ play a role in this metric. As the ϕ_0_ term represents signal propagation time which is treated as a constant, this term is ignored. This metric is visually represented in the right plot of Figure 3: as all the vectors are now the same length, successful detection of the signal only occurs when 95% of the k trials are within the highlighted part of the circle. The subtle difference between formula C and formula D is when the averaging was done: in formula C, the averaging was done as the first step for the vector mean and the second step for the scalar mean. This formula is used by Norcia and Tyler (1984), and they derived it from first principles. Formula D, on the other hand, executes the averaging at the very last step. This formula is used in EEGLAB (Delorme and Makeig, 2004), in Mitra and Pesaran (1999), Kamphuisen et al. (2008), and Derzsi (2017) as well. The coherency can also be calculated from the variances of the phases across trials: $1-\frac{\zeta_{k}}{\xi_{k}}$, but this is often impractical because it is computationally more intensive to get the phase angle variances of *V*_*k*_ when compared to formula C and D.

We are not aware of any studies which compares the performance of different metrics that can be used for analysing SSVEPs. Norcia and Tyler argue that the vector mean amplitude (B) is preferable to the scalar mean (A), “since noise voltage is proportional to the square root of the bandwidth in Hz” and so “the resulting improvement in signal-to-noise is equal to sqrt(n)” (Norcia and Tyler, 1984). One can see from Equation (3) that if the phase of the original response were entirely random, i.e., ζ_*k*_ were uniformly distributed between [0°360°], the first complex component would tend to average to zero across many repetitions, and this metric would asymptote to *A*_0_, the mean amplitude of the signal. However, in general, which metric is best must depend on the properties of the signal as well as of the noise and thus is hard to derive a priori. In this paper, we find out which of these four metrics provide the most reliable results with the least number of trials to detect weak signals such as the ones used in frequency tagging studies. We compare the performance of the four metrics shown in Table 1 using a simulation and the SSVEPs from a frequency tagging experiment where human subjects viewed temporally modulated stereoscopic disparity (Norcia and Tyler, 1984; Derzsi, 2017).

## 2. Methods

We created two simulations in Matlab to evaluate our four metrics in Table 1, and we also replicated Norcia and Tyler's single-carrier frequency tagging study (Norcia and Tyler, 1984).

### 2.1. Simulations

We created two simulations: In the first simulation, we created two different SNR conditions, for the purpose of finding out how many trials are required for our four metrics to successfully detect the signal. The second simulation, we approximated how many trials are needed to reliably detect the signal as a function of the SNR.

The distributions in both simulations are built up from three components: white noise (1); an interfering “birdie” signal (2), which is an 8 Hz sine wave. This signal is unstable, its frequency and its phase angle are both randomized across trials. The purpose of the birdie signal is to imitate an independent separate oscillation in the EEG signal, similarly to alpha waves for example. In both simulations, we use the four metrics to find the frequency tagged carrier (3), which is a weak 13 Hz sine wave, and is always in the same phase across the trials. The detection criterion is always the same: the Fourier component belonging to the frequency tagged signal has to be above the noise threshold, which is the 95*th* percentile of the distribution created from the birdie signal and the white noise in the frequency band of 0.1–30 Hz.

#### 2.1.1. First Stimulation: Performance of Metrics for a Fixed SNR

In the first simulation, the noise had a peak value of 1, the birdie had the peak value of 8. In the “Strong” condition had the peak value of the frequency-tagged signal was 0.042, which corresponds to an SNR of 0.047. The “Weak” condition had a much smaller frequency-tagged signal, with the peak value of 0.003, which corresponds to an SNR of 0.0003. In each iteration, the simulation code creates a new data set with an increasing number of trials, and calculates the probability of the signal being part of the noise distribution. The signal is deemed to be successfully detected for each metric when this probability is less than or below 0.05.

#### 2.1.2. Second Simulation: Number of Trials Required as a Function of SNR

The second simulation, the noise had a peak value of 0.825, the birdie had a peak value of 0.175, and the SNR was varied between 0.1 and 0.00063 in 10 logarithmic steps. The signal was reliably detected with a metric, when the probability of the signal's Fourier component was above the noise distribution, with a probability of being part of the noise distribution (consisting of the white noise and the birdie) being <0.05. Since this simulation involves working with random numbers, the simulation is executed 20 times and the resulting trial numbers were averaged. To shorten the execution time, a maximum trial limit of 3,000 was set. If the number of trials exceeded this number without detecting the signal for a metric, no valid results was returned.

The Matlab code used to create the simulation results are included as Supplementary Material to this paper.

### 2.2. EEG Experiment

#### 2.2.1. Participants and Ethics

As part of a PhD project (Derzsi, 2017), we measured the spatio-temporal limits of depth perception. As a secondary experiment in the project which is essentially a replication of Norcia and Tyler's study, we collected 537 good trials from the EEG recordings of 4 participants (adults, 2 males, 2 females, age 23.5 ± 3.5 years). The project was approved by the Ethics Committee of the Faculty of Medical Sciences of Newcastle University.

#### 2.2.2. Stimulus and Trials

**Figure 4:**
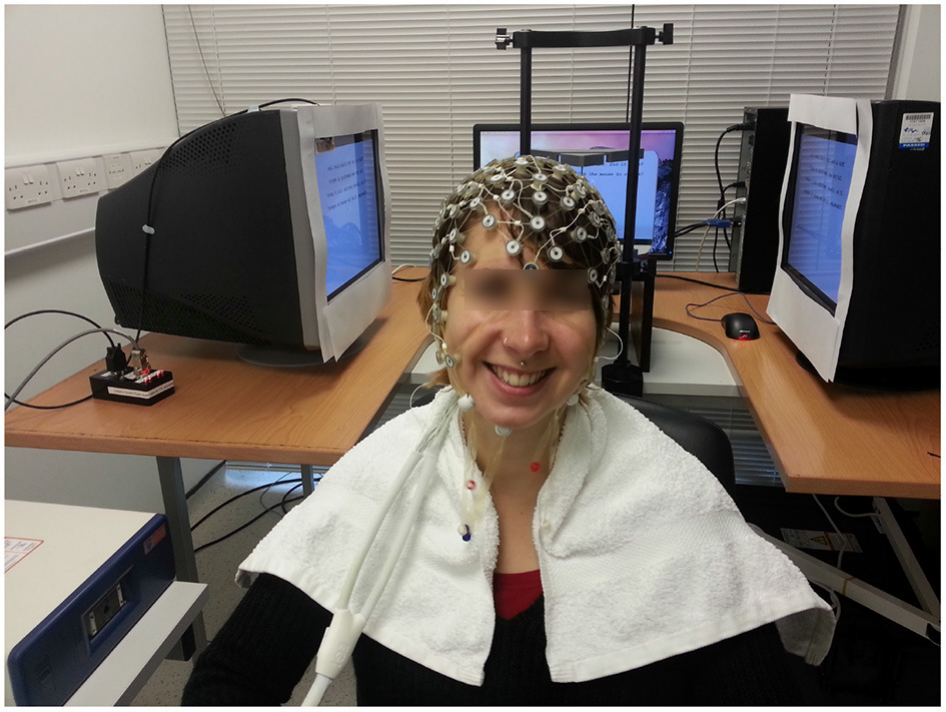
One cheerful participant wearing our 128-channel EGI electrode cap, sitting in front of the 3D display. The mirror assembly of the Wheatstone stereoscope is just behind her head.

We used two calibrated Dell P992 CRT monitors in a Wheatstone stereoscope configuration to create our stereoscopic stimulus. The participant's head was placed on a chin rest in front of the mirror, and the displays covered 40 × 40° visual angle. The refresh rate of the monitors was 100 Hz. A photo of the set-up is shown in Figure 4.

We wrote a stimulus using Psychtoolbox (Pelli, 1997; Kleiner et al., 2007) which displayed a dynamic random-dot stereogram (Julesz, 1971), consisting of an equal number of black and white dots, presented on a 50% gray background. The mean luminance of the stimulus was 57.5 *cd*/*m*^2^, and the dot density was 0.06%. The locations of the dots were updated at every frame (10 ms).

**Figure 5:**
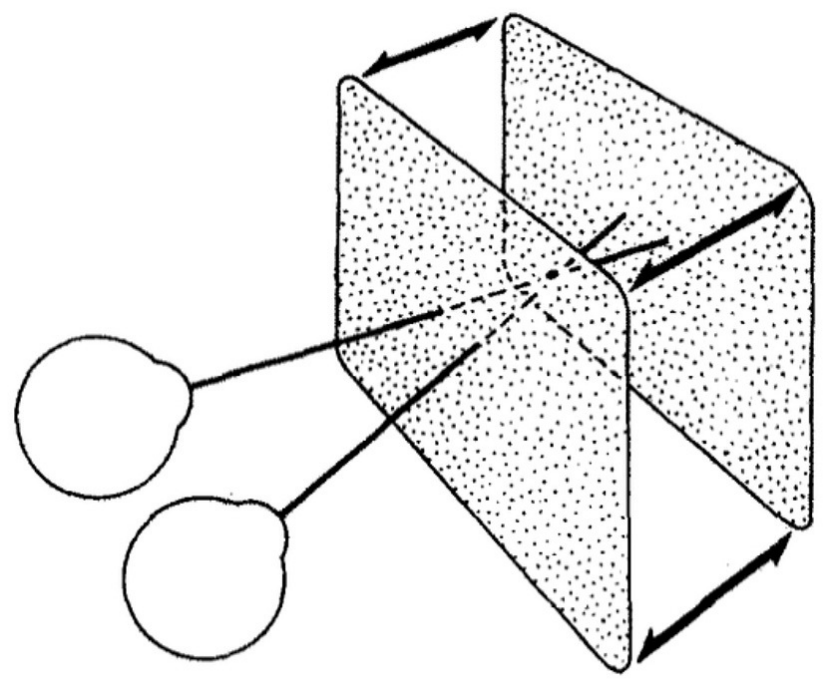
The stimulus used in our experiment and in Norcia and Tyler's experiment: a correlated random-dot stereogram plane that bounced in and out of the screen plane with positive and negative binocular disparity. Norcia and Tyler used a modified television set to create anaglyph stereograms, our experiment used two CRT monitors in a Wheatstone stereoscope arrangement (Norcia and Tyler, 1984). Copyright 1984, with permission from Elsevier.

**Figure 6:**
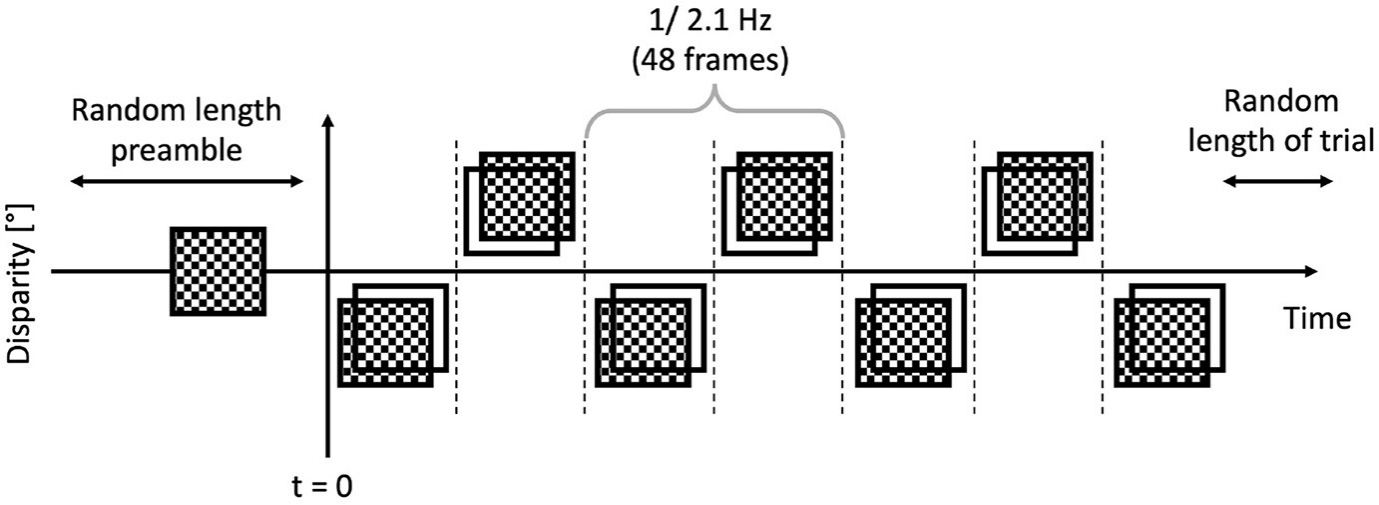
Anatomy of a trial: the preamble was displayed for a random time between 1 and 1.5 s, then the modulated bouncing disparity appeared on the screen, for a random time between 5 and 6 s. The timing of the “disparity onset” event was recorded with millisecond precision.

The trials were executed by the participants, and they were short, between 6 and 8 s. Each trial featured a “dot onset” preamble of between 1 and 1.5 s where the dots were displayed with zero disparity. Then once this time had elapsed, the applied binocular disparity (“disparity onset”) alternated between ±0.05°, at a rate of 2.1 Hz, or 48 frames, as depicted in Figure 5. This alternation continued for a random time between 5 and 6 s. The EEG traces were then temporally aligned such that the onset of the disparity alternation occurred at *t* = 0 as per Figure 6 corresponded.

#### 2.2.3. EEG Recordings and Preprocessing

We used Electrical Geodesics' (“EGI,” Eugene, Oregon, USA) 128 channel HydroCel Geodesic Sensor Net (GSN) system to record our EEG data. The electrode cap is connected to the participant using silver chloride electrodes, with sponges soaked in an electrolyte, which is made of saline solution with baby shampoo mixed in. For each channel, the impedances were kept below 50 kΩ. The signal was sampled at 1 kHz, and the “disparity onset” event was presented as a TTL signal that was directly coupled from the CRT monitor using a photodiode and a peak detector circuit.

In Net Station (EGI's proprietary EEG software) we filtered the continuous recordings between 0.1 and 70 Hz, and a narrow band-stop (notch) filter was also in place to reduce the effect of the 50 Hz mains hum. The recordings then were segmented to the “disparity onset” event within the trials, and further processing was done in Matlab. Trials containing cardiovascular artifacts, or eye blinks and other muscle movements were rejected. If a trial had more than 10% noisy channels that showed signs of electrode detachment, or the drying of electrolyte for example in the EEG signal, it was also rejected. For further analysis, we only used a single channel (no. 72 of the GSN), which was located just above the inion.

#### 2.2.4. Analysis

We analyzed the trials using our own code in Matlab, and some analysis was done using EEGLAB (Delorme and Makeig, 2004). We analyzed the simulation results and the EEG data the same way, with the exception that we investigated only the first harmonic of tagged frequency in the simulation.

In the spectral analysis, the neural response to the stimulus is detected by identifying a peak at the known temporal frequency of the stimulus, or a harmonic. In both spectral metrics (formula A and B in Table 1), we compared the sample's Fourier component value at these signal harmonics to every other frequency (i.e., the noise) in the analysis. We counted successful signal detection as occurring when the value at the harmonic is larger than the 95th percentile of the noise. The probability of false detection is calculated by the ratio of how many other peaks in the noise are above the 95th percentile, and how many Fourier components are included at distinct temporal frequencies in the analysis:

(4) $$\begin{array}{l} p=\left(\sum_{f=f_{min}}^{f_{max}}S_{signal}(f)>[95th\;percentile(N_{noise}(f))]\right)\\ \;/\left(\sum \left(f_{max}-f_{min}\right)\right) \end{array}$$

where *S*(*f*) is the value of the signal sample, *N*_*noise*_ noise distribution at the frequency *f*.

*S*_*signal*_(*f*) is always a single component in the simulation. In the experimental data analysis, we used the first six harmonics of the temporal frequency of the periodic stimulus.

#### 2.2.5. Analysis and Statistics on our EEG Data

In the trials, we looked at the $\left(1/\sqrt{f}\right)$-compensated spectrum and the calculated coherency of the SSVEPs of one single channel at the central occipital area. Since the waveform of the temporal modulation of the stereogram's depth plane is a symmetrical square wave which only contains odd harmonics, and we know that the neural mechanism triggered is sensitive to changes in disparity, we expect the first derivative of this signal to be present in our EEG recordings. Therefore, we only consider the presence of the even harmonics to be linked to processing, and the odd harmonics to be the original signal passing through the human visual system.

The coherency values are compared against a large number (1,000) synthesized, phase-scrambled noise data sets. Unlike the bootstrapping operation, where the data would be re-sampled at a trial level, we generated our data sets with identical number of trials to the real data we analyzed. This allows us to calculate the 95*th* percentile of the noise distribution not just across the spectrum, but across data sets, and create a reliable measure of upper noise floor or “noise threshold.” If a coherency value is above this noise threshold, we know immediately that it is statistically significant. The exact probability can be worked out using formula 4 as well.

## 3. Results

### 3.1. Simulations

#### 3.1.1. First Simulation: Performance of Metrics for a Fixed SNR

**Figure 7:**
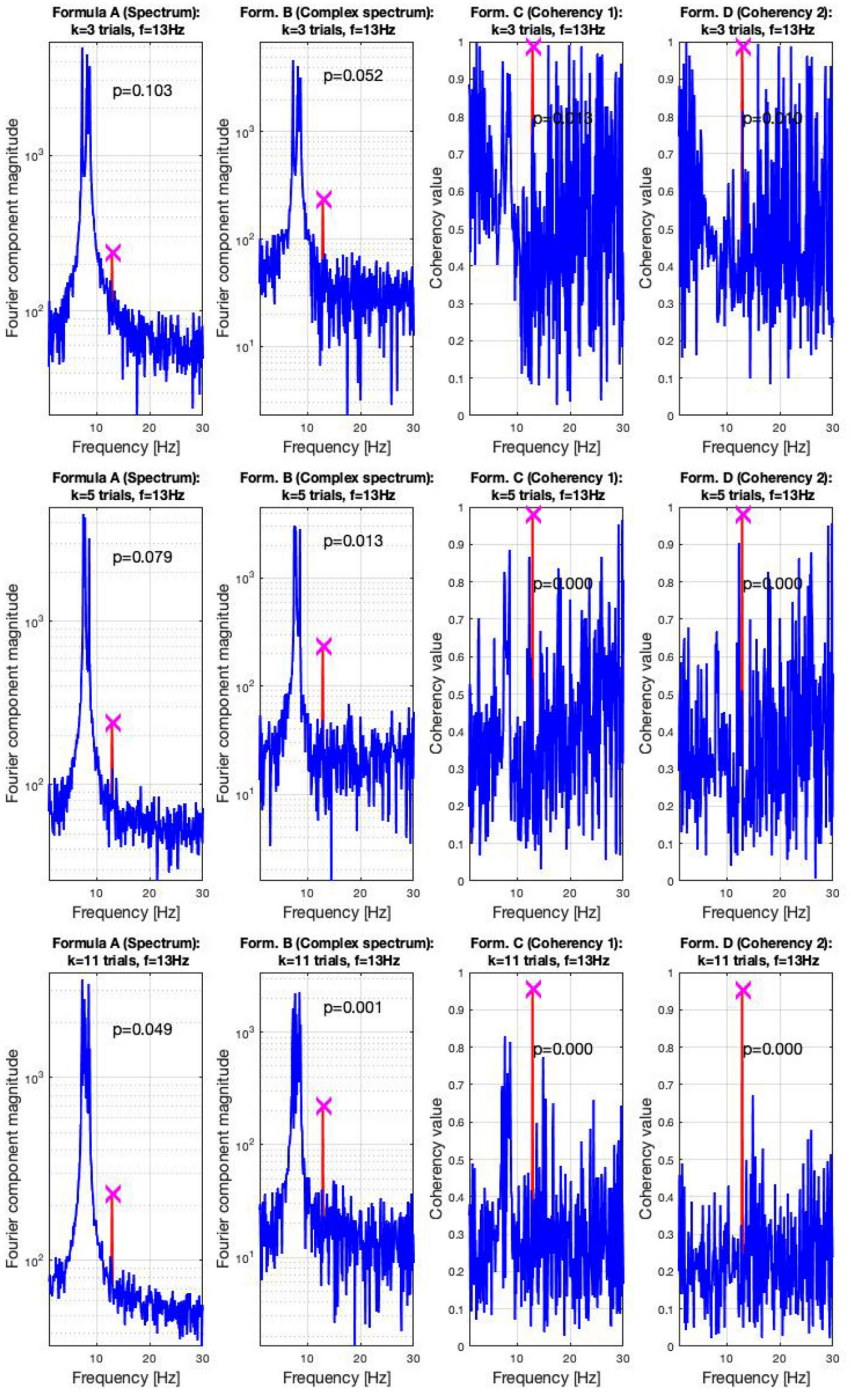
In the high SNR (0.0047) condition tested by the simulation, only a handful of trials were enough to detect the hidden signal: in the top right plots, formula C and D only required three trials to achieve reliable detection. In the middle plots, in the middle left, the complex spectrum detected the signal at 4 trials. For the spectrum to do the same, 11 trials were necessary, as visible in the bottom plot. These plots are generated using the code in the Supplementary Material, using the same signal. The frequency tagged signal is at 13 Hz, and the unstable birdie signal originating from an independent oscillation is at 8 ±0.8 Hz.

In the high SNR (0.047) condition, the signals are shown in three sets of plots in Figure 7. In this condition, the frequency-tagged signal is so powerful that the coherency analysis (formula C and D in Table 1) is capable of detecting it with only three trials. This is shown in the top middle and top right plot. The complex spectrum (formula B in Table 1) is on the edge of detecting the signal too, but it's *p*-value is slightly below the significance threshold of 0.05. However, just by adding an extra trial in the middle plot, it also successfully detects the frequency tagged signal. While there is a distinct peak at the spectrum (formula A in Table 1) at the tagged frequency of 13 Hz as well, it takes far more, 11 trials for the spectral analysis to detect it reliably. This is shown in the bottom left plot.

For the low SNR (0.0003) condition, the performance of each metric (see **Figure 15**) under the conditions set in the included supplementary code show the probability of erroneous detection (i.e., significance level, *p*-value) as a function of how many trials are included in a data set. The coherency analysis (formula C and D in Table 1) requires around 200 trials for a *p*-value of 0.05, with formula C showing to be a little more sensitive than formula D. The complex spectrum metric (formula B in Table 1) requires about 800 trials to achieve the same significance level. For the same signal and same conditions, the spectral analysis (formula A in Table 1) does not provide meaningful results. For the purpose of illustration, **Figure 15**'s scatter of p-values are fitted with a simple exponential model (*y* = *a* × *exp*(*bx*)), with good quality fitting *r*^2^ ≥ 0.85, with the exception of the spectral analysis, as the *p*-values hover around 0.4–0.5.

In the low SNR condition, the signals are shown in the top plots of Figure 6 at 350 trials, and in the top plots at 900 trials. The spectral analysis shows in the far left plots how powerful the birdie signal is with respect to the tagged carrier signal: under these circumstances, detecting the tagged signal in the spectrum is impossible. In top middle left plot, the complex spectrum (formula B in Table 1) does show a peak at the tagged frequency, but its p-value is too high to be deemed reliable. The coherency analysis (formula C and D in Table 1, middle and far right plots) show confidence levels below 0.05, implying that the signal was reliably detected. Formula D's coherency value is lower (0.15) than Formula C's (0.2), but -similarly to the complex spectrum- formula C shows a false positive at the birdie signal's frequency of 8 Hz.

**Figure 8:**
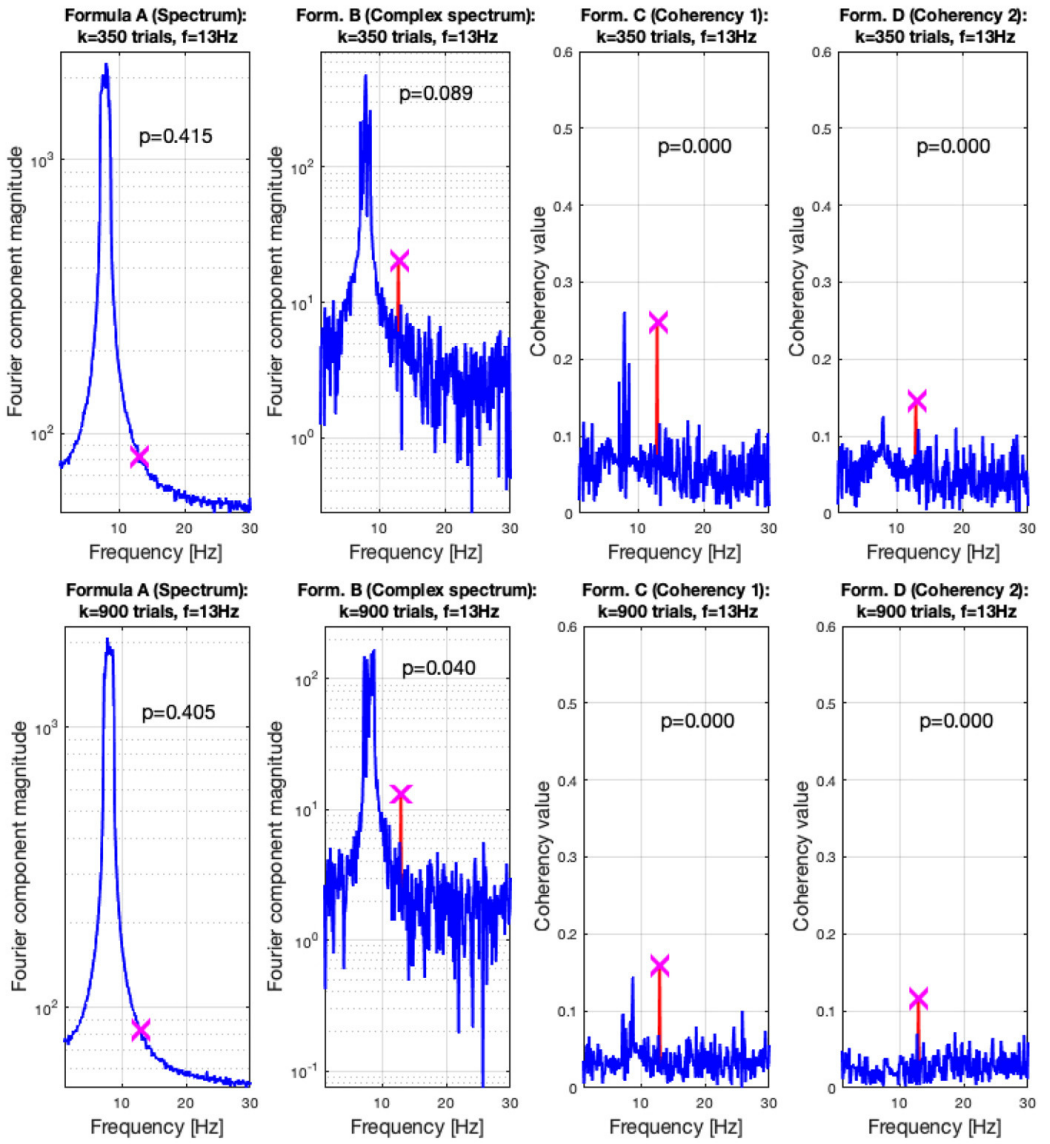
In the low SNR (0.0003) condition tested by the simulation, at 350 trials in the top plots, the hidden signal was detected successfully with the coherency (formula C and D in Table 1) method. The complex spectrum (formula B in Table 1) provided reliable results only at the bottom at 900 trials. The spectrum failed to detect the signal at all. These plots are generated using the code in the Supplementary Material, using the same signal. The frequency tagged signal is at 13 Hz, and the birdie signal originating from an independent oscillation is at 8 ± 0.8 Hz. The birdie signal's interference is visible in all plots except formula D's in the far right of the bottom plots.

Adding more trials to the experiment shows two additional benefits: from Figures 7, 8, the noise floor is noticeably lower, particularly with the coherency analysis at the right plots; and adding more trials to the data set allows the complex spectrum to detect the frequency tagged signal as well. The effect of the birdie is still visible in all metrics, but with formula D in the bottom far right plot of Figure 7 it is considerably diminished.

#### 3.1.2. Second Simulation: Number of Trials Required as a Function of SNR

In **Figure 14**, the straight lines are estimations from information theory. At high SNRs, up to about 0.06 and above, only a single trial is enough to find the frequency tagged signal with the spectrum and the complex spectrum techniques (formula A and B in Table 1). Below these SNRs, the required trial numbers increase rapidly, requiring about a hundred trials at the SNR of 0.03. The traces split noticeably at around the SNR of 0.02, where the spectrum requires about 4–5 times more trials than the coherency (formula C and D in Table 1) and the complex spectrum to detect the signal (B in Table 1). By the time the SNR gets to as small as 0.01, more than 3,000 trials are needed to be detected with the spectrum, at which point the simulation terminated. Going further to weaker and weaker SNRs, the complex spectrum and the coherency lines split: at around the SNR of 0.0035, approximately the complex spectrum requires double the number of trials than the coherency to detect the signal. This ratio diminishes somewhat as the SNR approaches even smaller values. At the SNR of 0.002, more than 3,000 trials are required for the complex spectrum to detect the signal, at which point the simulation terminates. Interestingly, the required number of trials seem to peak at the SNR of 0.002, and fewer trials are needed for worse SNRs. At the SNR of 0.0005, the complex spectrum required about 1,000 trials, whereas the coherency only needed 4–500.

### 3.2. EEG Experiment

**Figure 9:**
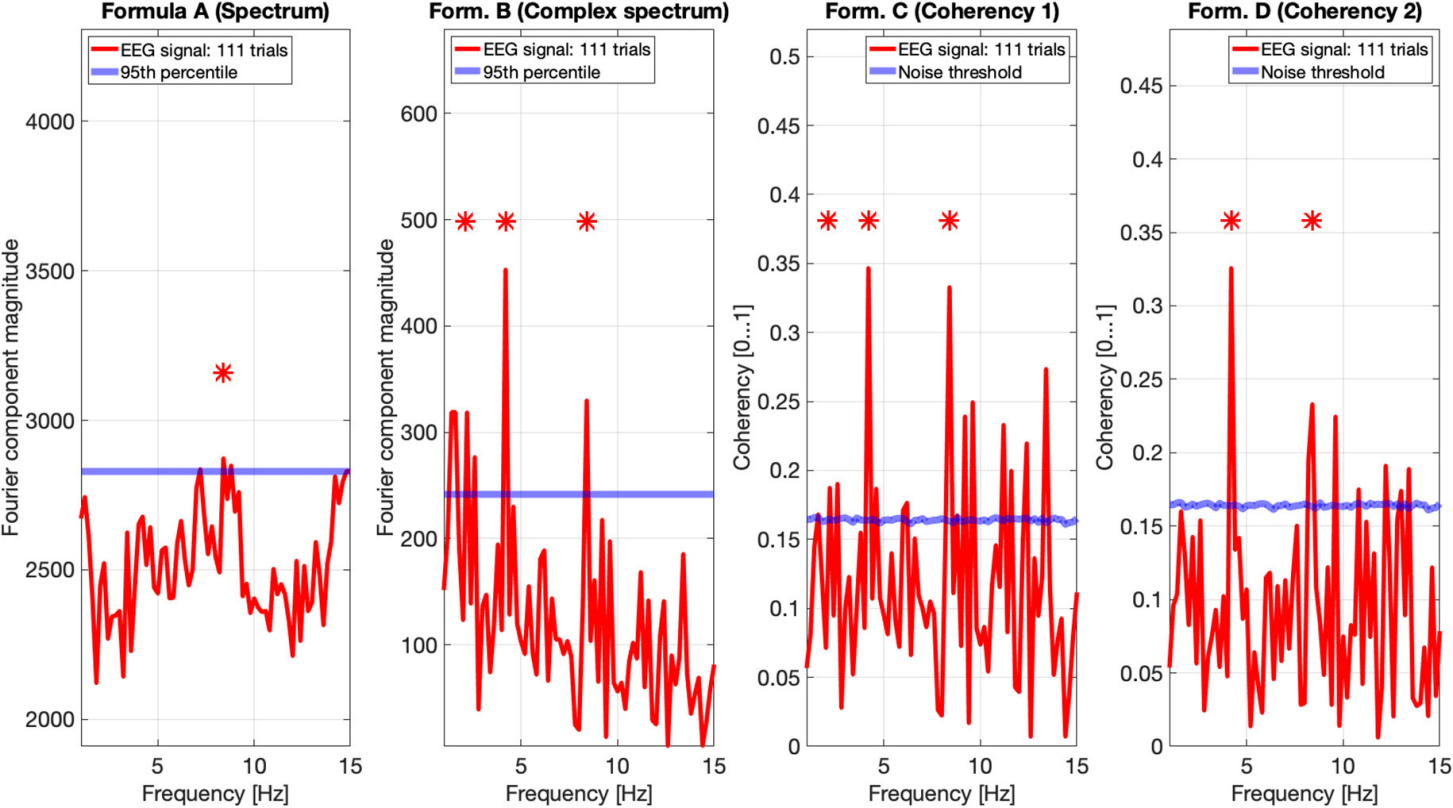
EEG data of participant AO. The spectrum (formula A in Table 1) does seem to show a peak above the 95*th* percentile at the fourth harmonic (8.4 Hz) of the temporal modulation frequency. The complex spectrum shows more peaks harmonics of the 2.1 Hz temporal modulation frequency. The coherency analyses (formula C and D in Table 1) in the right two plots do show significance at the second and fourth harmonics at the temporal modulation frequency. In the coherency analysis, the signal was compared against a generated white noise comparison standard, with identical number of trials. The blue “noise threshold” line indicates the *p* = 0.05 significance level, which is the 95^*th*^ percentile of our phase-scrambled synthesized noise distribution sets.

**Figure 10:**
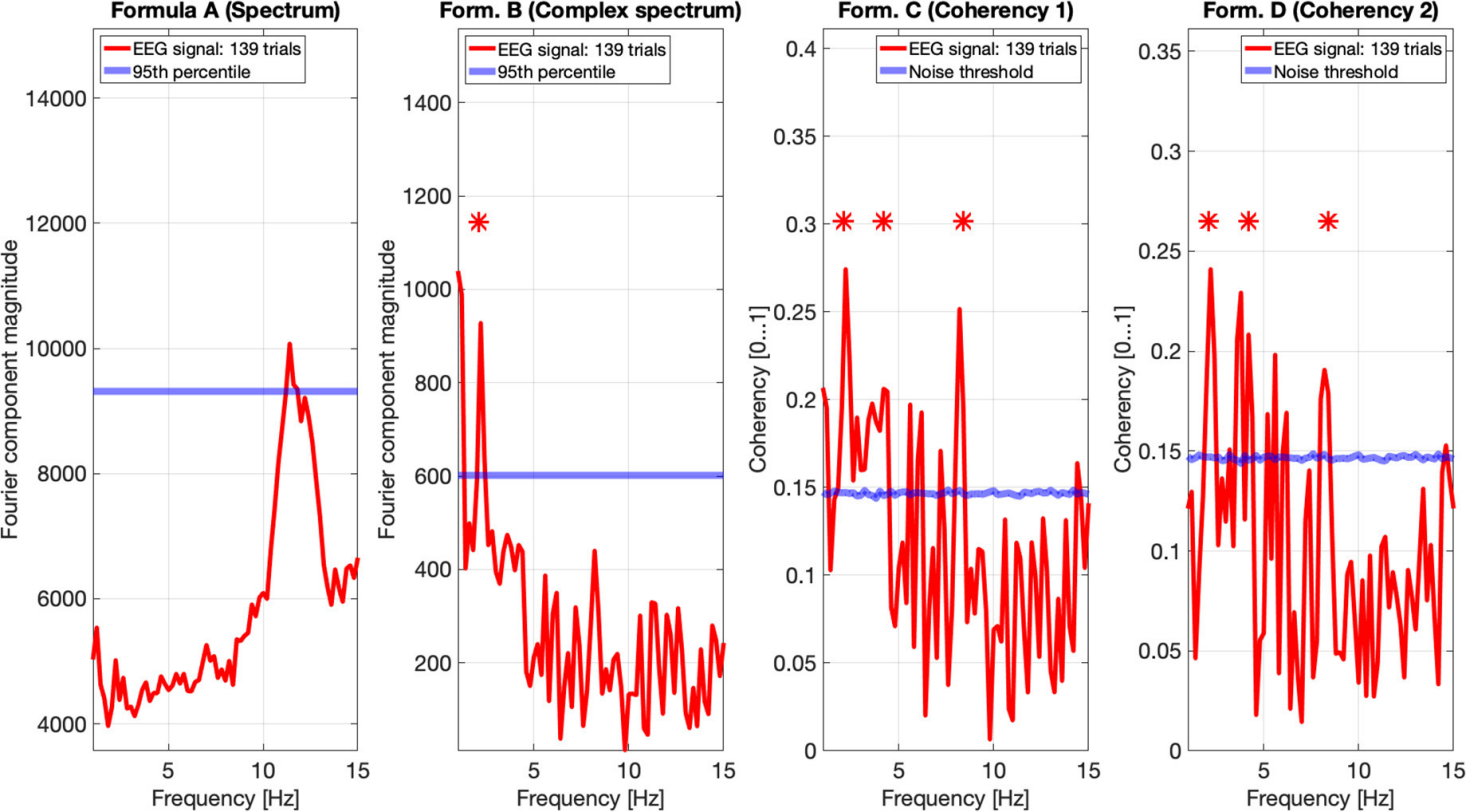
EEG data of participant SA. The spectrum (formula A in Table 1) in the far left plot shows elevated power in the Alpha band, but not at any harmonics of the temporal modulation frequency of the stimulus. The complex spectrum shows one peak at the first harmonic the 2.1 Hz temporal modulation frequency. The coherency analyses (formula C and D in Table 1) in the right two plots do show significance at the second and fourth harmonics at the temporal modulation frequency. In the coherency analysis, the signal was compared against a generated white noise comparison standard, with identical number of trials. The blue “noise threshold” line indicates the *p* = 0.05 significance level, which is the 95^*th*^ percentile of our phase-scrambled synthesized noise distribution sets.

Figures 9, 10 show the results of all four metrics (see Table 1), for two participants. The plots were generated from 111 and 139 trials, respectively. In the two left (spectrum and complex spectrum) plots, the blue lines are the 95*th* percentile of the noise spectrum with the harmonics of the stimulus signal excluded.

The spectrum (formula A in Table 1, far left plots), besides a peak around the alpha (8–12 Hz) band with one participant, is unremarkable. The near left plots show the results of the complex spectrum calculated with formula B in Table 1, which does seem to show peaks at the first harmonic of the temporal modulation frequency with both participants, and the second and fourth harmonics with one participant. There are other peaks present above the 95*th* percentile in unrelated frequencies between the first and the second harmonics in a similar fashion to the response to the birdie signal presented in the simulation results in Figures 8, 7, respectively.

The two right plots show the coherency of the signal (formula C and D in Table 1), with the peaks on the red traces highlighting the harmonics of the temporal modulation frequency. The blue lines in the right plots are noise thresholds, which is calculated as the mean 95*th* percentile of 1,000 phase-scrambled noise data sets, with matching number of trials to the EEG data. This “noise threshold” is used as an indicator for significance: if a signal is above the noise threshold, it is deemed to be significant, and thus the signal is detected. The star above the peak indicates that the first, second, and fourth harmonics are distinct from the noise, with a detection error probability of <0.05. The exact probabilities are calculated with formula 4. Furthermore, the coherency peaks are smaller with formula D than formula C, but at the same time there are fewer coherency peaks above the noise threshold at unrelated temporal frequencies.

**Figure 11:**
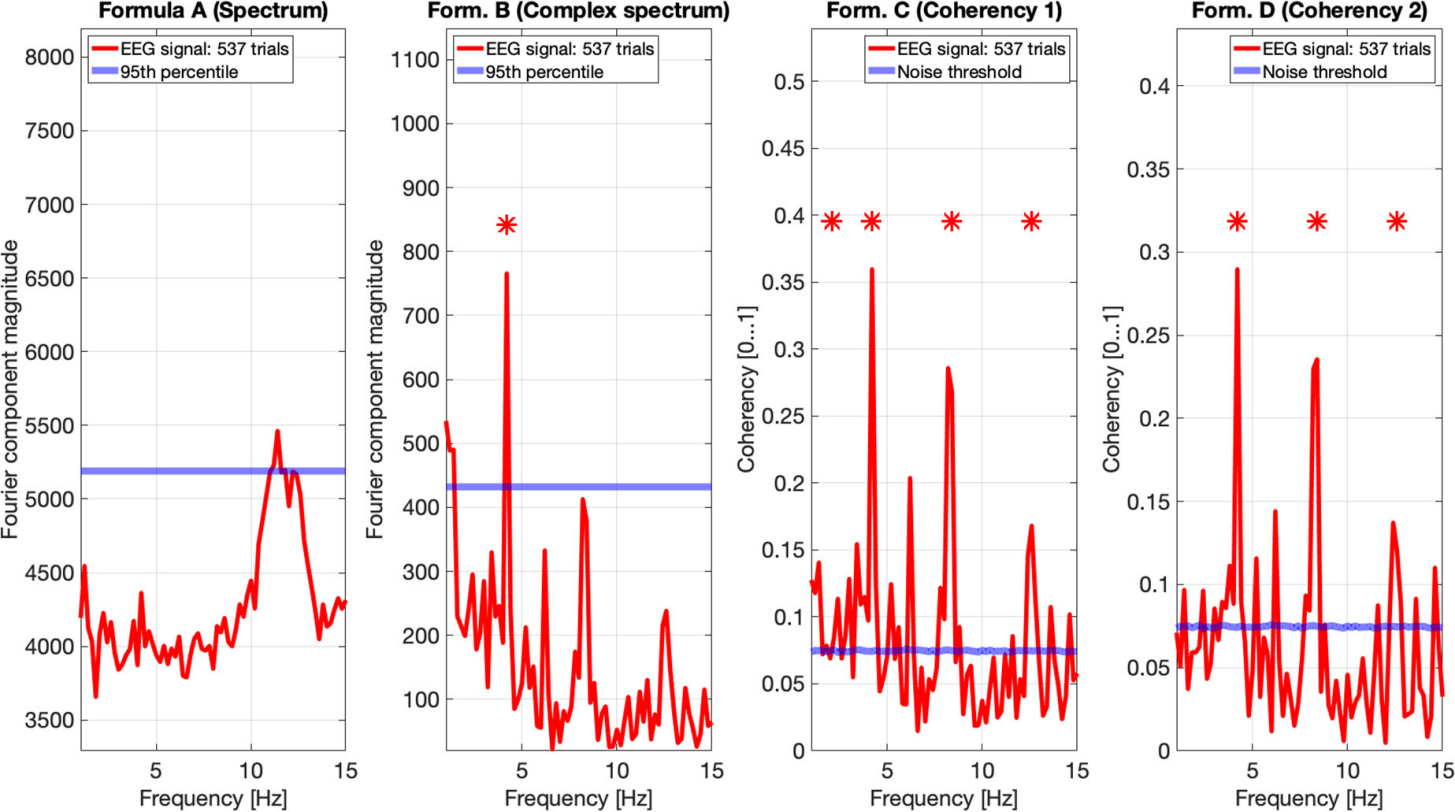
In this figure EEG data from our four participants is pooled together at a trial level creating a single data set with 537 trials. We can see that the spectrum (formula A in Table 1, far left plot) does not show any significant peaks at any of the harmonics of the stimulus frequency. However, it shows elevated spectral power in the Alpha (8–12 Hz) band. The complex spectrum (formula B in Table 1, middle left plot) does detect the second harmonic (4.2 Hz) of the stimulus frequency, and possibly the fourth (8.4 Hz) harmonic as well, but it's just below the 95*th* percentile threshold line. In contrast, the coherency analysis (formula C and D in Table 1, right plots) detects the first six harmonics of the stimulus, with formula C in the middle right plot additionally detecting the first harmonic as well. When compared with the same plots of Figures 9, 10, the coherency values only slightly reduced while the noise threshold lowered considerably. This implies that the trial-level data pooling may be a worthy trade-off to improve sensitivity with the coherency analysis.

In Figure 11, we pooled together the trials of our four participants, and plotted the results from all four metrics: the spectrum (formula A in Table 1) is in the far plot, the complex spectrum (formula B in Table 1), and the coherency (formula D in Table 1) are in the right plots. The spectrum of the EEG recordings show the alpha band of 10...12 Hz increased. Apart from this, the spectrum's plot is unremarkable, there are no visible peaks at any of the harmonics of the depth alternation frequency. The complex spectrum in the near right plot did detect the second harmonics of the stimulus frequency, and there are other distinct peaks at further harmonics, but below the significance threshold.

However, the two left plot's coherency analysis shows distinct peaks at the second (4.2 Hz), fourth (8.4 Hz), and sixth (12.6 Hz) harmonics, which are phase-locked to the stimulus stereograms's depth alternation. Additionally, formula C also detected the base harmonic (2.1 Hz) of the stimulus as well.

## 4. Discussion

### 4.1. EEG Experiment: Successful Replication of Norcia and Tyler's 1984 Study

**Figure 12:**
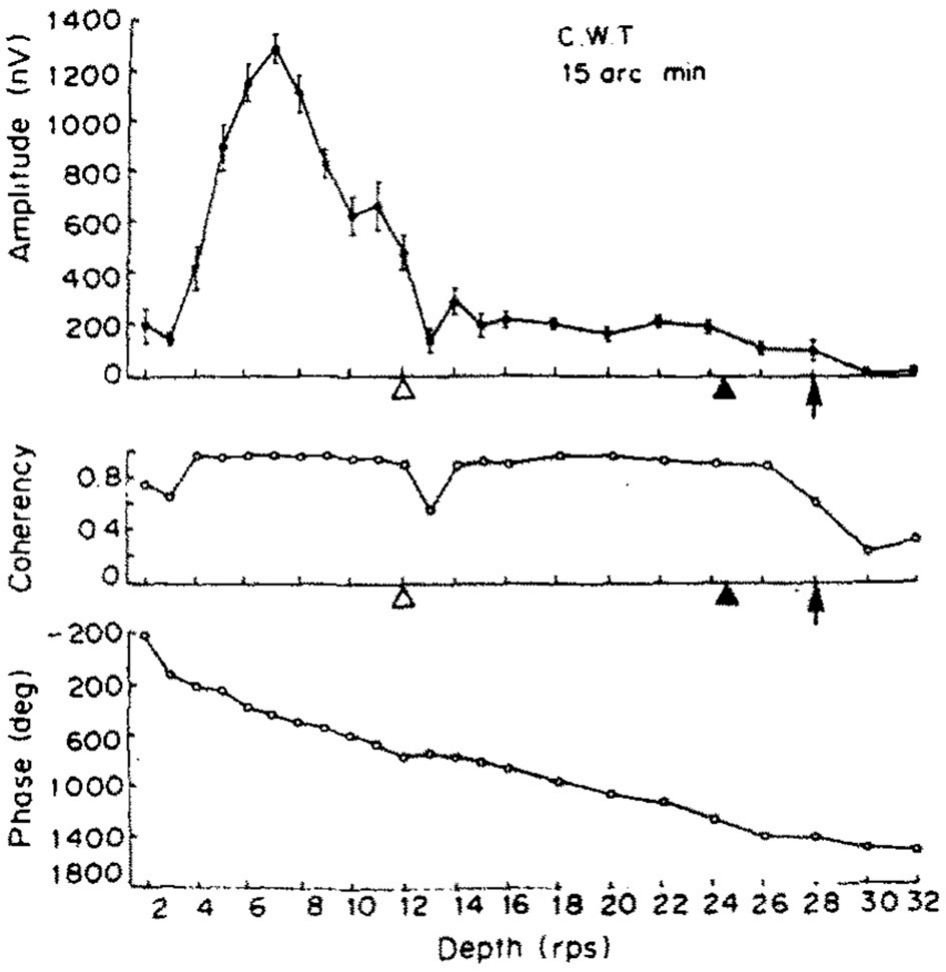
The results of one of the two participants in Norcia and Tyler (1984). **(Top)** The amplitude of the acquired EEG signal at the center occipital area, showing a peak response between 2 and 4 Hz depth alternation frequency. **(Middle)** Measured coherency (formula C in Table 1) value. **(Bottom)** The phase angle plotted of the signal, implying that there is a constant or near-constant propagation delay between the depth alternation and the recorded EEG signal at the second harmonic of the depth alternation frequency. Copyright 1984, with permission from Elsevier.

While we aimed to replicate the original (Norcia and Tyler, 1984) study as closely as we could in our experiment, since some hardware could not be obtained easily over 30 years after the original study. We have implemented some changes: we doubled the number of participants to 4 and they were naïve to the subject, we rejected trials based on detected artifacts in the EEG signal instead of letting the participants report bad trials themselves, and a single temporal frequency was chosen for the depth alternation which provided us with the strongest neural response. Our display covered a larger visual angle and did not require the wearing of anaglyph glasses. Unlike our study with constant stimulus frequency, Norcia and Tyler used the frequency sweeping technique for the depth alternation. Their frequency response of a single participant is shown in the top plot of Figure 12, with a peak at around 3.5 Hz. The second harmonic of our temporal modulation frequency was reasonably close to this value, 4.2 Hz.

Apart from the above, our dot density and the peak disparity values were close to identical to the original study. Norcia and Tyler concentrated only at analysing the second harmonic of the depth alternation frequency, because they used discrete tunable filters on the recorded EEG waveform and they calculated the coherency value manually.

Our signal acquisition and processing was done using computers, and so were the time-frequency transforms, which allowed us to not only investigate coherency at the second harmonic of the temporal modulation frequency, but to do so over the entire spectrum until the 500 Hz Nyquist-limit. However, we only analyzed and plotted a smaller sensible part of this band, from 0.1 to 15 Hz. Perhaps a notable drawback of using discrete time signals is when analysing short bursts of it: in our case, only 5 s after the disparity onset event mean that the window of the Fast Fourier Transform is rather small, which lead to a relatively poor, but yet still acceptable frequency resolution of 0.2 Hz per Fourier component. Without increasing the sampling frequency or using longer trials, this is unavoidable. We also believe that this is one of the reasons why our coherency values are smaller than what Norcia and Tyler reported. However, with our statistics, the frequency tagged signal is reliably detected, and thus we have successfully replicated Norcia and Tyler's study.

### 4.2. Control Measures

**Figure 13:**
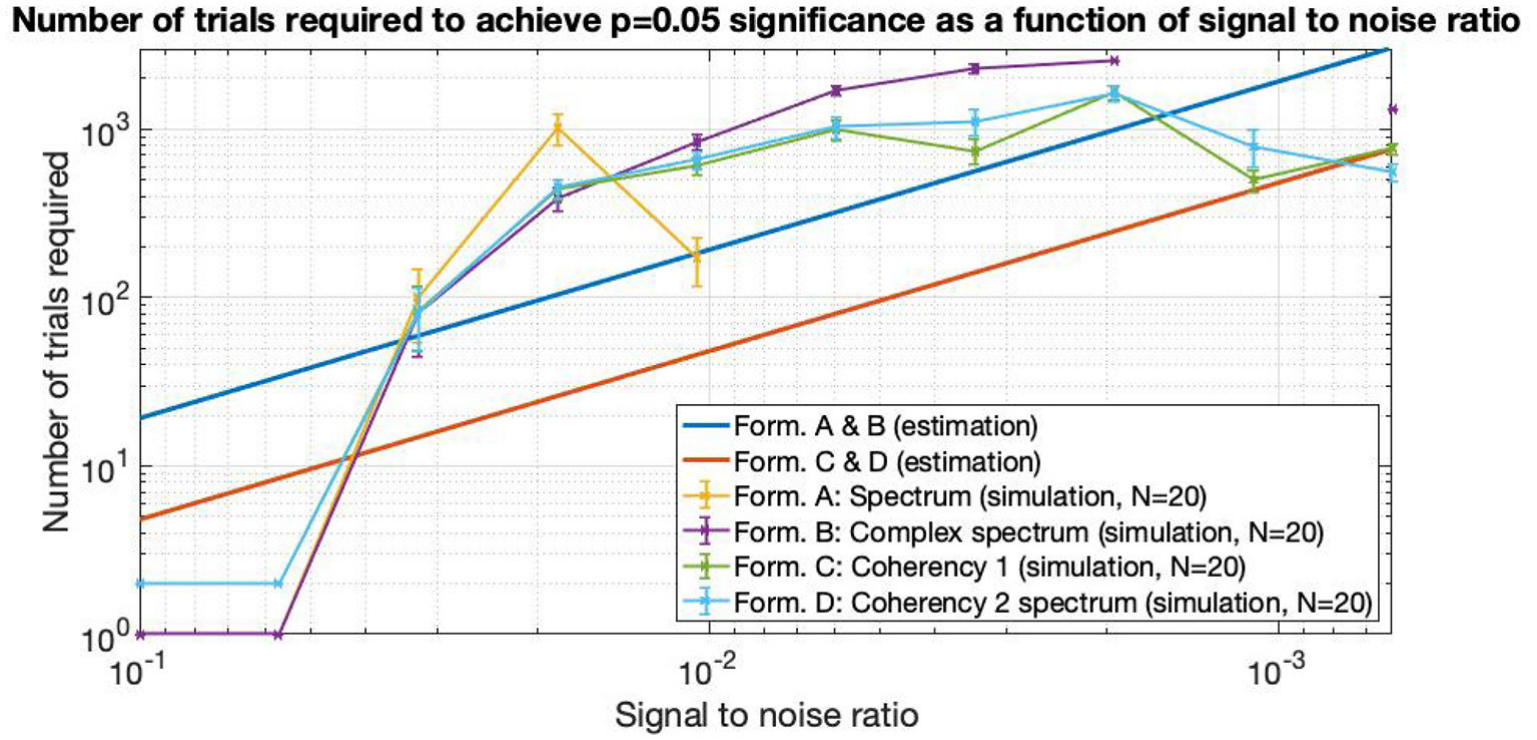
Coherency values at different uniform plane (0 cycles-per-degree grating) conditions in Derzsi (2017): the gray line is the temporal modulation frequency of the grating, and the colored (red, blue, magenta) lines are the (second, fourth, and sixth, respectively) harmonics. As the temporal modulation frequency increases, the second harmonics' coherency value follows. The “generated noise coherency” is used as the comparison standard to detect the presence of the signal.

The data presented here is a subset of a larger PhD project (Derzsi, 2017), where we used this technique to characterize depth perception in the human visual system: we have found the corresponding coherency peaks for conditions with different temporal and spatial frequencies, but for the purpose of this paper that compares various metrics in a frequency tagging experiment, only a small fraction of results are included here. In Figure 13, we demonstrate that the coherency peak follows the temporal modulation frequency, and that the signal is detectable with fewer trials.

### 4.3. Can Pooling Across Participants Ever Be a Sensible Choice?

A good practice in studies is to collect a number of trials from a participant, generate a per-individual result, and then pool across them to come up with the grand average that is used in the final analysis.

Pooling across participants at a trial level when analysing the spectrum does not produce meaningful results, because of the risk of data being driven by a small number of powerful outliers, which can lead to improper conclusions.

However, since the coherency analysis rejects the amplitude component of the EEG data by principle, the risk described above is eliminated. As each participant will have a different signal propagation time in their brains, the phase angles of the frequency-tagged signals will be different as well.

Pooling across participants at a trial level will result in a reduced coherency value because of the increased spread of the phase angle distribution of the signal (different ϕ_0_ and different ζ_*k*_ in Equation 3 for each participant). Since in the coherency analysis, we are evaluating the coherency data against synthesized phase-scrambled control data sets, the actual coherency value itself does not matter: as long as it's significantly elevated from the noise, it is detected successfully. In our case, this is shown in Figures 9–11: the coherency value for a single participant at 4.2 Hz is 0.33, which reduces to 0.28 after pooling four participants together. However, the noise level is 0.18 in the single participant's data reduces to around 0.08 in Figure 11, which means that our chances of detecting the signal has increased despite the overall reduction of the coherency values, making pooling across participants at a trial level a worthwhile trade-off for low temporal frequencies. Provided that the variance of signal propagation times of individuals is less than half a period of the temporal frequency of the stimulus, the overall reduction of coherency values will not be considerable, and this minimizes the risk of losing the signal.

### 4.4. Noise Model Choice

In nature, the electrical noise is fundamentally pink noise: the noise power follows a 1/*f* pattern in the spectrum, and the EEG signals are no exception. Since the EEG equipment measures voltage and not power, the noise follows a $1/\sqrt{f}$ pattern. This has to be compensated for when evaluating the spectrum and the complex spectrum (formula A and B in Table 1). However, irrespective of what type of “noise” we are dealing with—whether external electrical noise or the electrical signal of some unrelated biological function—the noise will not be phase-locked to the temporal modulation frequency of the stimulus. Thus, following a Fourier transform, the distribution of the arguments of the vectorial representations of the complex Fourier components are completely stochastic within the interval of 0 and 360°. Additionally, since in a frequency tagging experiment we consider every irrelevant signal component as noise, it is difficult to create a model that accurately imitates the signals created by the brain. In the simulation, the presence of white noise and the birdie signal was an attempt to replicate this, but it is fundamentally insufficient. In reality, there may be a large number of birdies present, mixed with transients and other artifacts from various sources. For instance, at 111 (see Figure 9) and at 139 trials (see Figure 10), we should see some relevant peaks at the spectrum, even if they are below the significance threshold. The lack of peaks in the spectra of real data show that the noise model used in the spectrum simulations is too forgiving, and this leads to a rather optimistic prediction of the performance of the spectrum in the simulations. This, however, only applies to the spectrum, and not for the coherency analysis.

Since the coherency analysis effectively removes the amplitude component along with its noise component of the signal completely, the resulting phase noise distribution will always be a uniform distribution, irrespective of what type of noise the acquired signal contained. This property enables it to be compared against artificially generated controls with very good accuracy.

When comparing the coherency plot of the EEG signal in Figure 11 in between harmonics (for example 10 and 12 Hz, or 15 Hz and above in Derzsi (2017) with the coherency plots on either Figure 7 or Figure 6, we can see that the coherency values are indeed uniformly distributed with respect to temporal frequency.

We could have implemented any other noise types in the simulation, but for the sake of simplicity and to due to the fact that the coherency analysis is insensitive by principle to the type of noise used, we decided to use only white noise.

### 4.5. Comparing the Performance of Spectrum and Coherency With Weak Signals

The metric that performs the worst is the spectrum (formula A in Table 1). At very low SNRs the signal is undetectable with conventional spectral analysis. Findings from information theory (Proakis, 2000; Proakis and Salehi, 2008; Derzsi, 2017) suggest that techniques using or exclusively relying on the phase information perform more reliably at low SNRs. We have verified this with both our simulation results in **Figure 15** and even with real data in Figure 11. This further reinforces that spectral evaluation in a weak-signal frequency-tagging study is one of the worst things to do.

The best performer is the coherency analysis, formula C and D in Table 1: since it effectively rejects the amplitude component of a vector along with its noise component, it increases the SNR, and this property makes it less prone to external interfering signals. Since the coherency is a measure of how consistent the phase angles are across the trials, the actual phase angles themselves are not being taken into account. Rather, their distribution with respect to the whole circle is the property that carries information (see Figure 3, middle and right plots), and this makes the coherency analysis is far more resistant against noise than any other approach presented in this paper. The only extra information required in the stimulus is the annotation of the phase as well as the frequency: without it, the coherency analysis is useless.

**Figure 14:**
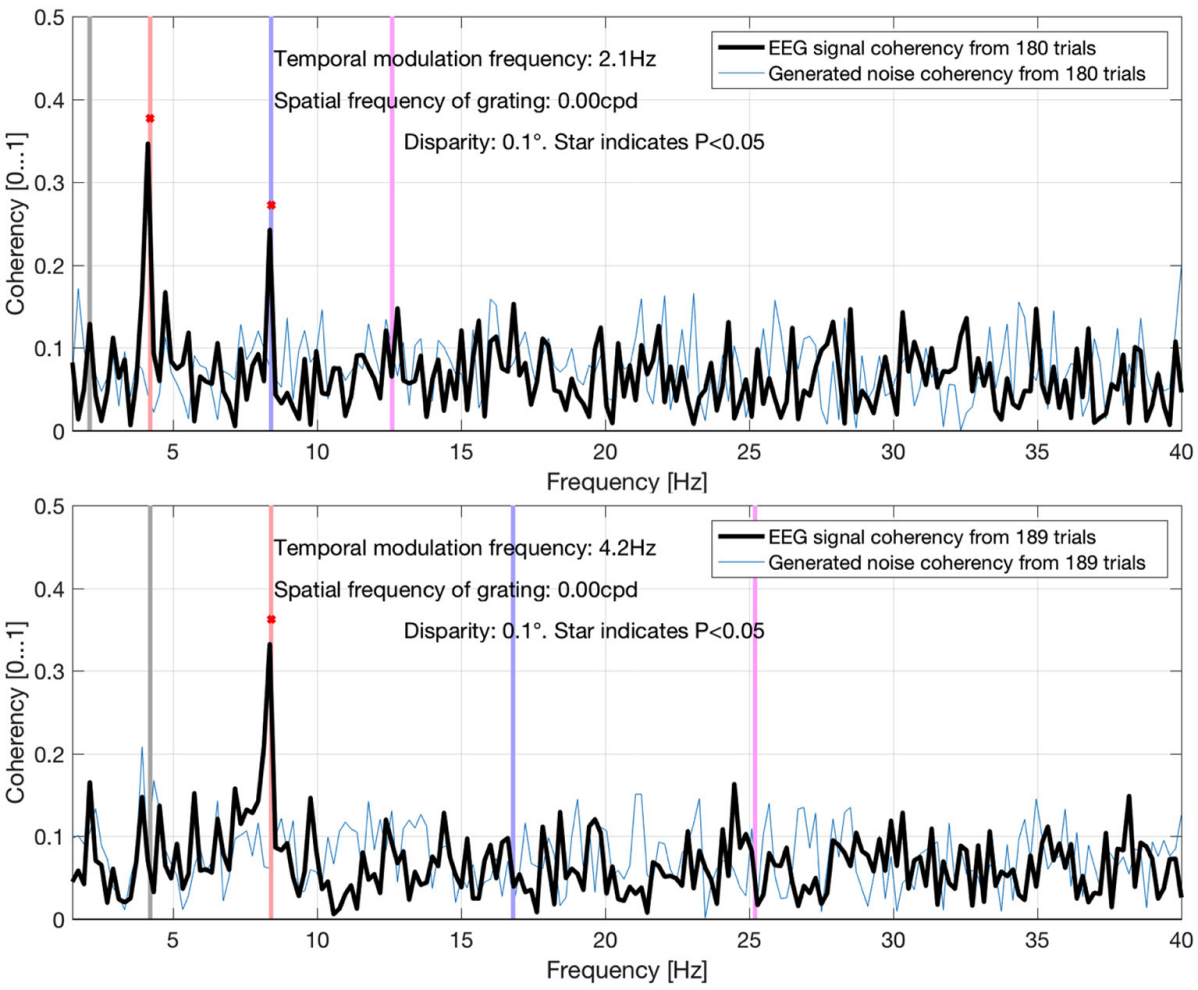
As the SNR gets worse and worse, more and more trials are needed to detect the signal. The estimations are from Equations (5) and (6), they are approximated from information theory. The simulated performance is shown for each formula. For SNR 0.06 and above, only 2 trials are enough to detect the frequency tagged signal for the coherency analysis and a single trial for the spectral analysis. Where the lines are incomplete, more than 3,000 trials were not enough to detect the signal, at which point the simulation was terminated. Having the simulation executed 20 times, we see that the coherency analysis requires the least number of trials to reliably detect the signal.

**Figure 15:**
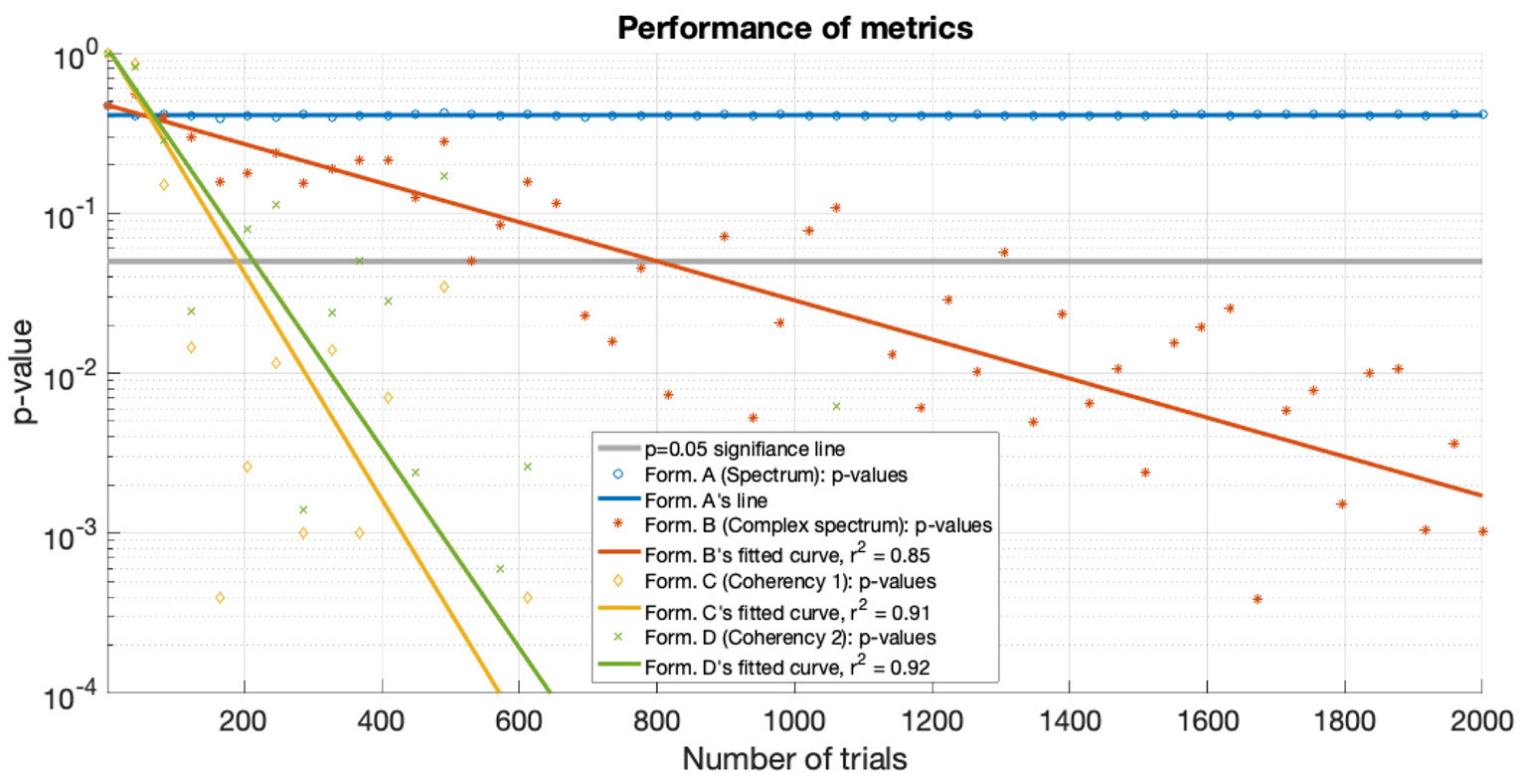
For the low SNR (0.0003) condition the *p*-values of the statistical analysis of the simulation is plotted against the number of trials used. Zero *p*-values are not shown due to the logarithmic axis. With the tagged signal being much weaker than the noise, the coherency analysis (formula C and D in Table 1) detects it with around 300 trials. For the same confidence level, about 800 trials are required for the complex spectrum (formula B in Table 1). At these low SNRs, the conventional spectral analysis (formula A in Table 1) completely fails to detect the tagged signal. The lines are fitted with a simple exponential model (*y* = *a*×*exp*(*bx*)), except for the spectrum, which hovers around 0.5.

Based on findings from information theory, we can derive the error performance (Proakis and Salehi, 2008) of various information enclosure (modulation) methods as a function of the SNR. This way, we can approximate how many trials are required as a minimum for successfully detecting a frequency-tagged signal (Derzsi, 2017) that has the phase information annotated with the error probability of *p* = 0.05. These are:

(5) $$L_{spectrum}=\frac{{\left[erfc^{-1}(2\times 0.05)\right]}^{2}]}{\text{SNR}}$$

(6) $$L_{coherency}\approx \frac{\frac{1}{4}\times {\left[erfc^{-1}(2\times 0.05)\right]}^{2}]}{\text{SNR}}$$

where *L* is the number of trials required, *erfc* is the complementary error function, and *SNR* is the signal-to-noise ratio. It is worth noting that these functions provide a strictly monotonically decreasing number of trials as a function of the SNR. These estimated performances are plotted in Figure 14, and provide similar results to the simulated performance in Figure 15: for example, at the SNR of 10^−3^, about 400 trials are required for coherency analysis and about 1,100 trials are required for the spectral analysis to provide meaningful results. When comparing this theoretical finding with the simulation results, it shows that these estimations show in Equations (5) and (6) are pessimistic at low SNRs, and optimistic at high SNRs. With a similar SNR in the simulation code that is included as Supplementary Material in this paper, these are about 4–500 and more than 3,000 trials, respectively.

Based on this information, provided that there are no external interfering signals and that the phase of the stimulus was known, we can improve a frequency tagged signal's detection probability by at least a factor of 4, just by analysing its coherency and not its spectrum. Of course, with real-world data this improvement is not as marked, but still considerable.

These equations that estimate the performance do not make a difference between the spectrum formula A and B and the coherency formula C and D. Spectral analysis either fails to detect a weak signal completely, or requires an unrealistically large number of trials to provide acceptable results. The spectral analysis method also is prone to show external interfering signals as false positive results. The coherency analysis, in all cases is a more sensitive approach for weak signals, with the capability of either greatly diminish or completely reject external interfering signals.

### 4.6. What SNR Is Reasonable in Real Data?

The actual observed SNR depends on the modality and the conditions of the stimulus. Bright flashes produce a very strong response, and relatively few trials are required to produce meaningful results. For example, Hébert-Lalonde et al. (2014) were able to find spatial visual deficits with a blinking spot on the screen from about just a minute of EEG recording. Binocular vision, on the other hand, produces a more subtle response: In Baitch and Levi (1988), over 100 trials were required to detect the lack of binocular visual processing in stereoblind participants. Binocular disparity processing produces even weaker signals: in the EEG study presented in this paper, more than 100 trials were required to detect the disparity-defined visual stimulus with the coherency analysis. Not even 500 trials were enough to detect the signal in the spectrum. Increasing the binocular disparity does not necessarily increase the strength of the neural response: too large, or too quickly changing disparities can not be fused properly. If the visual system does not have enough time to solve the stereo correspondence problem (Ip et al., 2014) or the participant is no longer able to follow it in depth (Alvarez et al., 2005), the depth perception from binocular disparity falls apart. This has been verified experimentally with psychophysics (Kane et al., 2014), and with frequency tagging (Derzsi, 2017) as well.

For spectral analysis, Equation (5) may be modified to provide an approximation of SNR from the number of trials used:

(7) $$\text{SNR}\approx \frac{{\left[erfc^{-1}(2\times 0.05)\right]}^{2}]}{L_{spectrum}}$$

This equation is approximate in nature, because many studies collect more data than what is absolutely minimally required for statistical significance. A visualization of this equation is shown with the dark blue straight line in Figure 14. In practice, this means that the estimation is somewhat pessimistic, so the SNRs reported by this formula are somewhat lower than in reality.

From the examples with flashing stimuli above, we estimate that the time-domain SNR are varying between 0.04 and 0.003: for 30 trials used in Hébert-Lalonde et al. (2014)'s flashing spot study is 0.04; for 100 trials used in Baitch and Levi (1988)'s binocular flash study is 0.02; 480 trials used in the control part of Nakanishi et al. (2018)'s flashing of characters is 0.003; 192 trials used in Gruss et al. (2012)'s flashing faces study is 0.015.

For the random-dot stereogram examples from above the SNR is worse, varying from approximately 0.01 to <0.002: for 100 trials used in Cottereau et al. (2012)'s disparity-defined annulus study is 0.015, but with the aid of fMRI; for 384 trials in Rideaux et al. (2020)'s moving circle defined by random-dot stereograms, it is 0.0025; in the study presented in this paper 537 trials were not enough to detect the frequency-tagged signal, therefore the SNR is estimated to be <0.004.

Therefore, the added sensitivity for the coherency analysis may be beneficial in these low SNR conditions, as it decreases the probability of erroneous detection. Additionally, since the coherency analysis is capable of detecting the signals in even lower SNRs, it will be an ideal analysis candidate for future or unpublished studies, where conventional analysis methods have failed provide convincing results.

### 4.7. Simulations

Both simulations demonstrate that the coherency analysis is the most sensitive method for detecting a weak signal in the SSVEP. In the first stimulation, where we used two conditions to imitate the presence of a strong (see Figure 7) and a weak (see Figure 6) signal, and in both cases, the coherency analysis required the lowest number of trials to detect the signal. This is further reinforced by the plot of performance in the low SNR condition in Figure 15, where the coherency analysis detected the signal at around 200 trials, the complex spectrum analysis detected the signal at around 800 trials, and the spectral analysis did not gain any confidence after 2,000 trials.

In the second simulation, where the required number of trials to achieve *p* = 0.05 significance are plotted against the SNR (see Figure 14), we see a similar picture: as the signal gets weaker and weaker, the sensitivity of the coherency analysis is more and more apparent. However, we must note that when the tagged signal is strong, and only a single trial is enough to detect it with the spectral analysis, it is pointless to do the coherency analysis, as it requires at least two trials to provide meaningful results.

From Equations (5) and (6), we know that the number of trials required is strictly monotonically increasing as the SNR decreases, but interestingly the behavior of the simulation results do not clearly show this. For example, prior to losing formula A's performance at the SNR of 0.01, it required only a fraction of trials to detect the signal than in the iteration before. The same is observed with the with the coherency results below the SNR of 0.002. We believe that this phenomenon is an artifact, due to the limit of double precision numbers, and the fact that we are rapidly approaching Shannon's theoretical limit of information capacity (Shannon, 2001) in these conditions. Realistically, considering that it is very difficult to collect more than 2–300 trials in a frequency tagging experiment from a single participant without fatigue, the practical limit of SNR at which the coherency analysis performs best is around *x* * 10^−3^. For the spectrum, this is considerably higher, *x* * 10^−2^. Therefore, the best use of the coherency analysis is when the signals are very weak, and could not be detected with any other method.

### 4.8. Does the Interfering Birdie Signal Matter at All in Coherency Analysis?

In a frequency-tagging study where the mechanism tested is well isolated, the straightforward approach to rely on the tagged frequencies themselves. Human stereopsis is a great example for such a mechanism, as it can do both intermodulation (Baitch and Levi, 1988) and frequency multiplication (Norcia and Tyler, 1984; Norcia et al., 2015) very cleanly, so the temporal frequencies in the analysis can be calculated easily. In these cases, a powerful unrelated signal can safely be ignored, and the more sensitive formula C may be used to find even the weakest signals.

However, when the operation of the mechanism studied is not so straightforward, such as the case with muscle movements (Nazarpour et al., 2012) or face perception (Boremanse et al., 2013), the experimenter may not have the luxury of ignoring any signal by labeling it as a birdie. In these cases, where finding and eliminating birdies is vital to avoid erroneous conclusions, formula D is the safer option as it's the most robust against external interference. We also suggest the use of a time-frequency analysis method in addition to the coherency analysis in such cases.

## 5. Conclusion

When employing the EEG frequency tagging technique in an experiment and analysing the SSVEP, spectrum may be the obvious choice at first glance. Due to its simplicity, it is easy to write reliable analysis software. The apparent ease of use, however comes at a price: as it preserves both the amplitude and phase noise components, spectral analysis is a very insensitive analysis method. Provided that the frequency and the phase information of the stimulus is known either by starting the stimulus in the same phase or recording the phase angle of it in each trial, it is possible to analyse the inter-trial coherency of the recordings, which can detect signals that are too weak to be seen in the spectrum. An added benefit is that the coherency may reliably be compared against artificially generated controls. Therefore, based on our simulation results and experimental verification, we found that the coherency analysis offers the detection of weaker signals, or requires fewer trials in an experiment.

Based on our analysis, we suggest the annotation of the phase angle of the stimulus and the use of coherency analysis instead of spectral analysis in future frequency tagging studies.

## Data Availability Statement

The original contributions presented in the study are included in the article/Supplementary Material, further inquiries can be directed to the corresponding author/s.

## Ethics Statement

The studies involving human participants were reviewed and approved by Ethics Committee, Faculty of Medical Sciences, Newcastle University. The patients/participants provided their written informed consent to participate in this study. Written informed consent was obtained from the individual(s) for the publication of any potentially identifiable images or data included in this article.

## Author Contributions

ZD designed and constructed the experimental hardware, collected data, wrote the signal processing script, and wrote the paper. The author confirms being the sole contributor of this work and has approved it for publication.

## Conflict of Interest

The author declares that the research was conducted in the absence of any commercial or financial relationships that could be construed as a potential conflict of interest.
