# Supplementary material for: Optimal Approach for Signal Detection in Steady-State Visual Evoked Potentials in Humans Using Single-Channel EEG and Stereoscopic Stimuli
Source: Front Neurosci. 2021 Feb 18;15:600543. doi: 10.3389/fnins.2021.600543 (PMC7935508; doi:10.3389/fnins.2021.600543)
Supplement: Supplementary file 1 [file Table_1.DOCX]

Supplementary material: simulation code.

Copy and paste the following code to Matlab.

% This script simulates frequency-tagged EEG. The same data is analysed

% via spectrum (formula A), complex spectrum (formula B), and coherency (formula C and D)

clear all;

close all;

clc;

%% Generate my signal.

sampling_rate = 1000; % In Hz.

trial_length = 10; % In seconds

samples = sampling_rate * trial_length; % How many samples are we working with?

% This is for white noise

carrier_amplitude = 0.003; % Super weak signal. (was 0.003)

frequency = 13; % This for the carrier and is in Hz.

% Create the carrier

carrier = carrier_amplitude * sind(frequency*(1:samples)*(360/sampling_rate)); %The divisor is to make time in seconds.

noise_amplitude = 1; %(was 1)

birdie_frequency_jitter_percent = 20; % This is frequency instability, in percent.

% This is for the birdie signal, which is an incoherent sinewave added to the noise.

birdie1_frequency = 8; % This is in Hz.

birdie1_amplitude = 3; % ...and this makes it a very weak signal. (Was 30)

% This birdie is disabled.

% This is an other birdie signal, that slows down from trial to trial.

birdie2_frequency = 29; % This is in Hz.

birdie2_amplitude = 0; % Stronger signal. Was 20.

birdie2_frequency_drift_per_second = -0.15; % This is in Hz per second.

birdie2_frequency_drift_per_trial = -0.0005; % This is in Hz per trial

%% Now organise things into trials.

% For a specific trial range, change this interval. De

trials_start_end = linspace(1, 2000, 50); % Trial numbers are linearly spaced.

%trials_start_end = logspace(0, 3.3, 50); % Log space for p-value performance plotting

% No matter what interval we set up, let's use the least number of them.

trials_start_end = unique(round(trials_start_end));

% We will use the entire trial in our FFT, so

fourier_coeff_we_want = round(frequency / ((sampling_rate/2) / (samples/2)) );

% Specify which band of the spectrum we want to plot.

plot_band = [1, 30]; %This is in Hz.

% Sanity check

if(max(plot_band) < frequency || min(plot_band) > frequency)

fprintf('Plot band is [%d, %d]Hz, whereas plot frequency is %dHz.\n', plot_band(1), plot_band(2), frequency);

error('Signal frequency is outside plotting bands!')

end

if(max(plot_band) < birdie1_frequency || min(plot_band) > birdie1_frequency)

fprintf('Plot band is [%d, %d]Hz, whereas plot frequency is %dHz.\n', plot_band(1), plot_band(2), birdie_frequency);

error('Birdie 1''s frequency is outside plotting bands!')

end

if(max(plot_band) < birdie2_frequency || min(plot_band) > birdie2_frequency)

fprintf('Plot band is [%d, %d]Hz, whereas plot frequency is %dHz.\n', plot_band(1), plot_band(2), birdie_frequency);

error('Birdie 2''s frequency is outside plotting bands!')

end

fft_plot_boundaries = 1 + round(plot_band / ((sampling_rate/2) / (samples/2)) );

number_of_fourier_coefficients = fft_plot_boundaries(2) - fft_plot_boundaries(1);

figure('Position', [0, 400, 880, 400]) %these numbers are obscure, I am assuming a 720p screen, this makes sure it fits.

fprintf('Assembling simulated experiments with the following number of trials:\n');

%% Loop-de-loop

for(i = 1:length(trials_start_end))

%pause(0.5); % Slow things down so we can see it.

%% First of all, assemble the data set.

if(~mod(i-1, 20))

fprintf('\n') %This is just for convenience, adds sporadic new lines to the console.

end

fprintf('%4d, ', trials_start_end(i));

for(j = 1:trials_start_end(i))

%% Generate the noise and the trial data

white_noise = noise_amplitude * (rand(1, samples) *2) - 1; % Simple white noise.

% We will also insert the birdie1 signal. This is going to be out of phase in each trial, but it is rather strong.

birdie1_frequecy_unstable = birdie1_frequency + birdie1_frequency * (rand()*2-1) * ((birdie_frequency_jitter_percent/2) / 100); % This makes the frequency 'wobble' a bit. Like a real signal.

birdie1 = birdie1_amplitude * sind(birdie1_frequecy_unstable*(1:samples)*(360/sampling_rate) + rand(1)*360); % The last bit adds a random phase.

% Birdie 2 signal will show some small drift per trial.

birdie2_drifted_frequencies = (birdie2_frequency + (j-1)*birdie2_frequency_drift_per_trial) + linspace(0, trial_length, samples) * birdie2_frequency_drift_per_second;

birdie2_drifted_frequencies_unstable = birdie2_drifted_frequencies + birdie2_frequency * (rand()*2-1) * ((birdie_frequency_jitter_percent/2) / 100); % This adds an extra frequency instability

birdie2 = birdie2_amplitude * sind( (birdie2_drifted_frequencies .* (1:samples)*(360/sampling_rate) + rand(1)*360) ); % The last bit adds a random phase.

% Noise is everything we don't want: normal noise and interfering signals.

noise = white_noise + birdie1 + birdie2;

%This is memory leaky, but it's just a small demo, so we are OK

trial_data(j, :) = carrier + noise; % This is the signal with white noise

noise_data(j, :) = noise; % You can try different types of noise here, but only white noise is implemented.

%Also, while we are here, generate the Fourier-transform of this too!

temp = fft(trial_data(j, :)); %FFT, with negative frequencies left in the array.

trial_spectrum(j, :) = temp(2:(1+samples/2)); %We now got rid of the negative frequencies, and we get rid of the DC component.

clear temp;

%Re-use the temp variable for white noise, for the complex spectrum stats

temp = fft(noise_data(j, :)); %FFT, with negative frequencies left in the array.

noise_spectrum(j, :) = temp(2:(1+samples/2)); %We now got rid of the negative frequencies, and we get rid of the DC component

clear temp;

end

%With our generated data, we need to look at the frequency component of interest in the FFT stuff.

trial_spectrum_scalar_means = mean(abs(trial_spectrum), 1);

trial_spectrum_vector_means = abs(mean(trial_spectrum, 1));

noise_spectrum_scalar_means = mean(abs(noise_spectrum), 1);

noise_spectrum_vector_means = abs(mean(noise_spectrum, 1));

% Calculate coherency a'la Norcia and Tyler 1984

norcia_tyler_trial_coherency = abs( mean(trial_spectrum, 1) ./ trial_spectrum_scalar_means );

norcia_tyler_noise_coherency = abs( mean(noise_spectrum, 1) ./ noise_spectrum_scalar_means );

% Calculate coherency: normalise the vectors

normalised_trial_vectors = trial_spectrum ./ abs(trial_spectrum); %This is now a bunch of vectors with the length of 1.

normalised_noise_vectors = noise_spectrum ./ abs(noise_spectrum); %This is now a bunch of vectors with the length of 1.

% Calculate coherency: take the absolute value and mean of the normalised vectors

zoltan_trial_coherency = abs(mean(normalised_trial_vectors, 1));

zoltan_noise_coherency = abs(mean(normalised_noise_vectors, 1));

%% STATS

% We simply work out the probability based on the white noise distribution:

% We take the sum of values that are above the one we are testing (i.e. the 'chosen value')

% ...and divide by the length of the noise array.

% For the purpose of performance comparison, we also save these P

% values.

% 1: Spectral peak versus peak noise. This is a Gaussian distribution, so we can use the t-test

spectrum_chosen_value = trial_spectrum_scalar_means(fourier_coeff_we_want);

spec_p(i) = sum(noise_spectrum_scalar_means >= spectrum_chosen_value) / length(noise_spectrum_scalar_means);

[~, spec_p(i)] = ttest2(spectrum_chosen_value, noise_spectrum_scalar_means, 'Tail', 'right');

% 2: Complex spectrum versus pink noise. This doesn't really look like Gaussian,

% but it's also not uniform distribution.

complex_spectrum_chosen_value = trial_spectrum_vector_means(fourier_coeff_we_want);

[~, com_p(i)] = ttest2(complex_spectrum_chosen_value, noise_spectrum_vector_means, 'Tail', 'right');

% 3: Norcia and Tyler's coherency versus white noise distribution

coherency_nt_chosen_value = norcia_tyler_trial_coherency(fourier_coeff_we_want);

cohnt_p(i) = sum(norcia_tyler_noise_coherency >= coherency_nt_chosen_value) / length(norcia_tyler_noise_coherency);

% 4: Zoltan's coherency versus white noise distribution

coherency_chosen_value = zoltan_trial_coherency(fourier_coeff_we_want);

cohy_p(i) = sum(zoltan_noise_coherency >= coherency_chosen_value) / length(zoltan_noise_coherency);

% The same but with one-tailed unpaired T-tests.

% [~, spec_p(i)] = ttest2(spectrum_chosen_value, noise_spectrum_scalar_means, 'Tail', 'right');

% [~, com_p(i)] = ttest2(complex_spectrum_chosen_value, noise_spectrum_vector_means, 'Tail', 'right');

% [~, cohnt_p(i)] = ttest2(coherency_nt_chosen_value, norcia_tyler_noise_coherency, 'Tail', 'right');

% [~, cohy_p(i)] = ttest2(coherency_chosen_value, zoltan_noise_coherency, 'Tail', 'right');

%% PLOT!

% This is the spectrum

subplot(1, 4, 1)

plot(linspace(plot_band(1), plot_band(2), fft_plot_boundaries(2)-fft_plot_boundaries(1)+1), trial_spectrum_scalar_means(fft_plot_boundaries(1):fft_plot_boundaries(2)), 'r-', 'LineWidth', 2);

xlim(plot_band)

ylim([min(trial_spectrum_scalar_means(fft_plot_boundaries(1):fft_plot_boundaries(2))), max(trial_spectrum_scalar_means(fft_plot_boundaries(1):fft_plot_boundaries(2))*1.1)])

%Add vertical line for thre freuqency marker.

xlabel('Frequency [Hz]', 'FontSize', 14)

ylabel('Fourier component magnitude', 'FontSize', 14)

title(sprintf('Formula A (Spectrum):\n k=%d trials, f=%dHz', trials_start_end(i), frequency), 'FontSize', 12)

set(gca, 'YScale', 'log')

grid on;

hold on;

plot(linspace(plot_band(1), plot_band(2), fft_plot_boundaries(2)-fft_plot_boundaries(1)+1), noise_spectrum_scalar_means(fft_plot_boundaries(1):fft_plot_boundaries(2)), 'b-', 'LineWidth', 2);

%Add text for P-value

scatter(frequency, spectrum_chosen_value, 300, 'mx', 'LineWidth', 3); % Third argument is marker size

text(frequency, max(ylim)*0.5, sprintf('p=%.3f', spec_p(i)), 'FontSize', 14, 'HorizontalAlignment', 'left');

hold off;

% This is complex spectrum

subplot(1, 4, 2)

plot(linspace(plot_band(1), plot_band(2), fft_plot_boundaries(2)-fft_plot_boundaries(1)+1), trial_spectrum_vector_means(fft_plot_boundaries(1):fft_plot_boundaries(2)), 'r-', 'LineWidth', 2);

xlim(plot_band)

ylim([min(trial_spectrum_vector_means(fft_plot_boundaries(1):fft_plot_boundaries(2))), max(trial_spectrum_vector_means(fft_plot_boundaries(1):fft_plot_boundaries(2))*1.5)])

xlabel('Frequency [Hz]', 'FontSize', 14)

ylabel('Fourier component magnitude', 'FontSize', 14)

title(sprintf('Form. B (Complex spectrum):\n k=%d trials, f=%dHz', trials_start_end(i), frequency), 'FontSize', 12);

set(gca, 'YScale', 'log')

grid on;

hold on;

plot(linspace(plot_band(1), plot_band(2), fft_plot_boundaries(2)-fft_plot_boundaries(1)+1), noise_spectrum_vector_means(fft_plot_boundaries(1):fft_plot_boundaries(2)), 'b-', 'LineWidth', 2);

%Add text for p-value

scatter(frequency, complex_spectrum_chosen_value, 300, 'mx', 'LineWidth', 3); % Third argument is marker size

text(frequency, max(ylim)*0.5, sprintf('p=%.3f', com_p(i)), 'FontSize', 14, 'HorizontalAlignment', 'left');

hold off;

% This is Norcia and Tyler's coherency.

subplot(1, 4, 3)

plot(linspace(plot_band(1), plot_band(2), fft_plot_boundaries(2)-fft_plot_boundaries(1)+1), norcia_tyler_trial_coherency(fft_plot_boundaries(1):fft_plot_boundaries(2)), 'r-', 'LineWidth', 2);

title(sprintf('Form. C (Coherency 1):\n k=%d trials, f=%dHz', trials_start_end(i), frequency), 'FontSize', 12)

xlim(plot_band)

ylim([0 0.6])

xlabel('Frequency [Hz]', 'FontSize', 14)

ylabel('Coherency value', 'FontSize', 14)

grid on;

%Add text for p-value

hold on;

plot(linspace(plot_band(1), plot_band(2), fft_plot_boundaries(2)-fft_plot_boundaries(1)+1), norcia_tyler_noise_coherency(fft_plot_boundaries(1):fft_plot_boundaries(2)), 'b-', 'LineWidth', 2);

scatter(frequency, coherency_nt_chosen_value, 300, 'mx', 'LineWidth', 3); % Third argument is marker size

text(frequency, max(ylim)*0.8, sprintf('p=%.3f', cohnt_p(i)), 'FontSize', 14, 'HorizontalAlignment', 'left');

hold off;

% This is my coherency

subplot(1, 4, 4)

plot(linspace(plot_band(1), plot_band(2), fft_plot_boundaries(2)-fft_plot_boundaries(1)+1), zoltan_trial_coherency(fft_plot_boundaries(1):fft_plot_boundaries(2)), 'r-', 'LineWidth', 2);

title(sprintf('Form. D (Coherency 2):\n k=%d trials, f=%dHz', trials_start_end(i), frequency), 'FontSize', 12)

xlim(plot_band)

ylim([0 0.6])

xlabel('Frequency [Hz]', 'FontSize', 14)

ylabel('Coherency value', 'FontSize', 14)

grid on;

%Add text for p-value

hold on;

plot(linspace(plot_band(1), plot_band(2), fft_plot_boundaries(2)-fft_plot_boundaries(1)+1), zoltan_noise_coherency(fft_plot_boundaries(1):fft_plot_boundaries(2)), 'b-', 'LineWidth', 2);

scatter(frequency, coherency_chosen_value, 300, 'mx', 'LineWidth', 3); % Third argument is marker size

text(frequency, max(ylim)*0.8, sprintf('p=%.3f', cohy_p(i)), 'FontSize', 14, 'HorizontalAlignment', 'left');

hold off;

%Update the figure as plotting happens

drawnow;

end

% Clean up some variables to save memory. I only have 8 GB at the moment.

clearvars noise_data trial_data noise_spectrum trial_spectrum normalised_noise_vectors normalised_trial_vectors

fprintf('\nDone! Now plotting:\n'); % add new line at the end.

%% Plot p-value performance

% For Matlab's curve fitting tool, you can use:

% trials_start_end as your X values

% spec_p for the performance of spectrum (formula 1)

% com_p for the performance of complex spectrum (formula 2)

% cohnt_p for the performance of coherency 1 (formula 3)

% cohy_p for the performance of cohy_p (formula 4)

% This plot is noisy, it's commented out, but it shows how much better

% coherency is when compared to spectrum.

% For convenience, we are reshaping the vectors so Matlab's curve fits would work

trials_start_end = reshape(trials_start_end, length(trials_start_end), 1);

spec_p = reshape(spec_p, length(trials_start_end), 1); % Spectrum (Formula 1)

com_p = reshape(com_p, length(trials_start_end), 1); % Complex spectrum (Formula 2)

cohnt_p = reshape(cohnt_p, length(trials_start_end), 1); % NT coherency(Formula 3)

cohy_p = reshape(cohy_p, length(trials_start_end), 1); % ZD coherency(Formula 3)

formula_to_use = 'exp1'; % This should be complementary error function-based, but close enough.

figure;

line([trials_start_end(1), trials_start_end(end)], [0.05, 0.05], 'Linewidth', 4, 'Color', '#AAAAAA') % Significance line, gray.

hold on;

% Spectrum (Formula 1)

scatter(trials_start_end, spec_p, 30, 'MarkerEdgeColor', '#0072BD') % Show data points

[spec_fit_model, spec_fit_quality, ~] = fit(trials_start_end, spec_p, formula_to_use); % Create a fitting model, but this time it's bad.

spec_line = spec_fit_model(trials_start_end); % Create the fitted line.

plot(trials_start_end, spec_line, 'LineWidth', 3, 'Color', '#0072BD');

% Complex spectrum (Formula 2)

scatter(trials_start_end, com_p, 30, '*', 'MarkerEdgeColor', '#D95319'); % Show data points

[com_fit_model, com_fit_quality, ~] = fit(trials_start_end, com_p, formula_to_use); % Create a fitting model and evaluate it

com_line = com_fit_model(trials_start_end); % Create the fitted line.

plot(trials_start_end, com_line, 'LineWidth', 3, 'Color', '#D95319');

fprintf('r^2 value of complex spectrum (formula 2)''s fitted curve is %0.3f\n', com_fit_quality.rsquare);

% NT coherency (Formula 3)

scatter(trials_start_end, cohnt_p, 30, 'd', 'MarkerEdgeColor', '#EDB120'); % Show data points

% Sometimes, if there are a lot of zeros in the data, the fitting

% algorithm fails, In this case, we can exclude zeros.

%[cohnt_fit_model, cohnt_fit_quality, ~] = fit(trials_start_end, cohnt_p, formula_to_use, 'exclude', cohnt_p == 0); % Create a fitting model and evaluate it

[cohnt_fit_model, cohnt_fit_quality, ~] = fit(trials_start_end, cohnt_p, formula_to_use); % Create a fitting model and evaluate it

cohnt_line = cohnt_fit_model(trials_start_end); % Create the fitted line, all data

%cohnt_line = movmean(cohnt_p, 1);

plot(trials_start_end, cohnt_line, 'LineWidth', 3, 'Color', '#EDB120');

fprintf('r^2 value of coherency 1 (formula 3)''s fitted curve is %0.3f\n', cohnt_fit_quality.rsquare);

% ZD Coherency (Formula 4)

scatter(trials_start_end, cohy_p, 30, 'x', 'MarkerEdgeColor', '#77AC30'); % Show data points

% Sometimes, if there are a lot of zeros in the data, the fitting

% algorithm fails, In this case, we can exclude zeros.

%[cohy_fit_model, cohy_fit_quality, ~] = fit(trials_start_end, cohy_p, formula_to_use, 'exclude', cohy_p == 0); % Create a fitting model and evaluate it

[cohy_fit_model, cohy_fit_quality, ~] = fit(trials_start_end, cohy_p, formula_to_use); % Create a fitting model and evaluate it

cohy_line = cohy_fit_model(trials_start_end); % Create the fitted line,

%cohy_line = movmean(cohy_p, 5);

plot(trials_start_end, cohy_line, 'LineWidth', 3, 'Color', '#77AC30');

fprintf('r^2 value of coherency 2 (formula 4)''s fitted curve is %0.3f\n', cohy_fit_quality.rsquare);

xlim([trials_start_end(1), trials_start_end(end)])

ylim([1/samples, 1]); % If we use log axes, this can't be zero.

set(gca, 'YScale', 'log');

%set(gca, 'XScale', 'log');

grid on;

set(gca, 'FontSize', 20);

legend('p=0.05 signifiance line', ...

'Form. A (Spectrum): p-values', sprintf('Form. A''s line'), ...

'Form. B (Complex spectrum): p-values', sprintf('Form. B''s fitted curve, r^{2} = %0.2f', com_fit_quality.rsquare), ...

'Form. C (Coherency 1): p-values', sprintf('Form. C''s fitted curve, r^{2} = %0.2f', cohnt_fit_quality.rsquare), ...

'Form. D (Coherency 2): p-values', sprintf('Form. D''s fitted curve, r^{2} = %0.2f', cohy_fit_quality.rsquare), ...

'FontSize', 14, 'Location', 'south');

xlabel('Number of trials');

ylabel('p-value');

title('Performance of metrics');

%save('all_trials_done.mat');

fprintf('All done.\n')
